# Bipolar disorder-iPSC derived neural progenitor cells exhibit dysregulation of store-operated Ca^2+^ entry and accelerated differentiation

**DOI:** 10.1038/s41380-023-02152-6

**Published:** 2023-07-04

**Authors:** Tristen Hewitt, Begüm Alural, Manali Tilak, Jennifer Wang, Natalina Becke, Ellis Chartley, Melissa Perreault, Stephen J. Haggarty, Steven D. Sheridan, Roy H. Perlis, Nina Jones, Nikolaos Mellios, Jasmin Lalonde

**Affiliations:** 1 Department of Molecular and Cellular Biology, University of Guelph, Guelph, ON Canada; 2 Center for Quantitative Health, Center for Genomic Medicine and Department of Psychiatry, Massachusetts General Hospital, Boston, MA 02114 USA; 3 Department of Biomedical Sciences, Ontario Veterinary College, University of Guelph, Guelph, ON Canada; 4 Department of Neurosciences, University of New Mexico School of Medicine, Albuquerque, NM USA; 5 Present Address: Neuroscience Center, HiLIFE, University of Helsinki, Helsinki, Finland

**Keywords:** Neuroscience, Bipolar disorder

## Abstract

While most of the efforts to uncover mechanisms contributing to bipolar disorder (BD) focused on phenotypes at the mature neuron stage, little research has considered events that may occur during earlier timepoints of neurodevelopment. Further, although aberrant calcium (Ca^2+^) signaling has been implicated in the etiology of this condition, the possible contribution of store-operated Ca^2+^ entry (SOCE) is not well understood. Here, we report Ca^2+^ and developmental dysregulations related to SOCE in BD patient induced pluripotent stem cell (iPSC)-derived neural progenitor cells (BD-NPCs) and cortical-like glutamatergic neurons. First, using a Ca^2+^ re-addition assay we found that BD-NPCs and neurons had attenuated SOCE. Intrigued by this finding, we then performed RNA-sequencing and uncovered a unique transcriptome profile in BD-NPCs suggesting accelerated neurodifferentiation. Consistent with these results, we measured a slower rate of proliferation, increased neurite outgrowth, and decreased size in neurosphere formations with BD-NPCs. Also, we observed decreased subventricular areas in developing BD cerebral organoids. Finally, BD NPCs demonstrated high expression of the *let-7* family while BD neurons had increased miR-34a, both being microRNAs previously implicated in neurodevelopmental deviations and BD etiology. In summary, we present evidence supporting an accelerated transition towards the neuronal stage in BD-NPCs that may be indicative of early pathophysiological features of the disorder.

## Introduction

Bipolar disorder (BD) is a chronic mood disorder characterized by severe symptomatic cycling between periods of depression and mania/hypomania [1]. Twin studies support a strong genetic component to the etiology of BD [2, 3], and genome-wide association studies (GWAS) coupled with exome sequencing analyses have identified more than 30 distinct genetic variations to date that confer disease susceptibility [4–6]. Notably, specific ion channel components and signaling effectors have been implicated as risk genes like *CACNA1C* (Calcium Voltage-Gated Channel Subunit Alpha1 C), which encodes a subunit of the L-type voltage gated Ca^2+^ channel and is involved in neurodevelopment and synaptic activity [7] and *NCAN* (Neurocan) that codes for a secreted extracellular matrix-forming protein that modulates axon guidance and cell migration [8]. However, despite those tantalizing genetic connections specific cellular processes responsible for the manifestation and progression of BD remain elusive.

The discovery of disease mechanisms responsible for BD has progressed slowly in part due to the heterogeneity of clinical symptoms in patients, the complexity of the underlying genetics, and the lack of comprehensive animal models [9, 10]. Fortunately, the growing availability of patient-derived induced pluripotent stem cells (iPSCs) and methods to culture those as differentiated neurons in a monolayer fashion, or as three-dimensional (3D) brain organoids, is now offering new options to face this challenge [11, 12]. Research using iPSC-based models have uncovered several cellular processes suspected to play a role in BD, including impaired migration and neurogenesis [13, 14], altered expression of membrane receptor and ion channel subunits [15], and differentially expressed genes that associate with differentiation, axonogenesis, and developmental maturation [16, 17]. Together these studies suggest that deviations in neuronal excitability and connectivity, which are suspected to be part of BD pathophysiology and disease progression, may be in fact rooted in neurodevelopment.

**Fig. 1:**
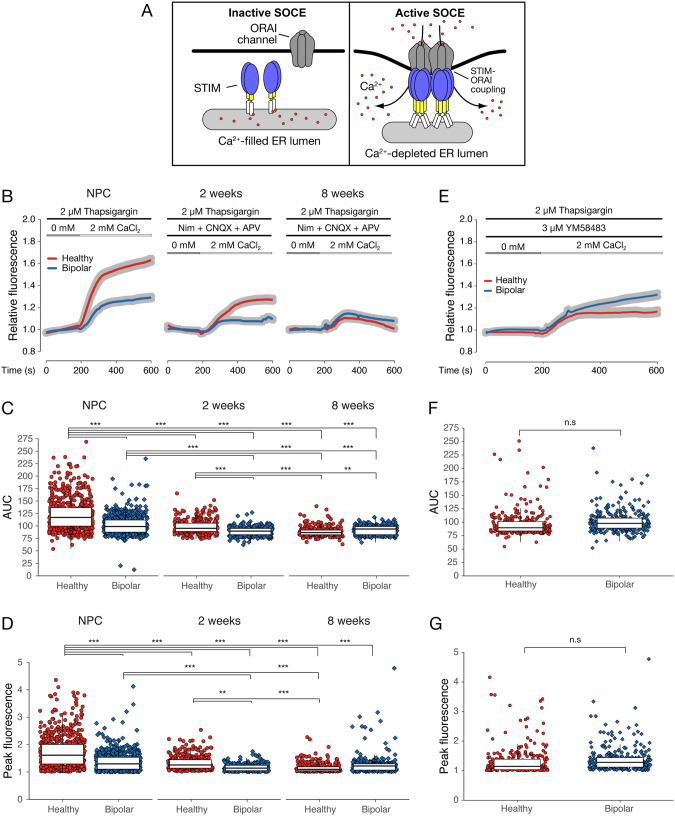
Ca^2+^ imaging reveals SOCE deficit in BD-NPCs and neurons. **A** A graphical representation of inactive (left) and active (right) SOCE. **B** Average increase in relative fluorescence (proportional to a baseline measurement from 0–180 s) of HC (red)- and BD (blue)-NPCs and neurons after Ca^2+^ re-addition. Data is presented as mean (red and blue curve) ± s.e.m (grey ribbon). **C** Area-under-the-curve (AUC) analysis from *t* = 185. Significant two-way ANOVA (F_7,2966_ = 139, *p* < 0.001) demonstrates that HC-NPCs (*n* = 598) had significantly higher AUCs than BD-NPCs (*n* = 595, *p* < 0.001), HC neurons differentiated for 2 weeks (*n* = 300, *p* < 0.001), BD neurons at 2 weeks (*n* = 287, *p* < 0.001), HC neurons at 8 weeks (*n* = 300, *p* < 0.001), and BD neurons at 8 weeks (*n* = 300, *p* < 0.001). BD-NPCs had significantly higher AUCs than BD neurons at 2 weeks (*p* < 0.001), HC neurons at 8 weeks, (*p* < 0.001), and BD neurons at 8 weeks (*p* < 0.001). HC neurons at 2 weeks had significantly higher AUCs than BD neurons at 2 weeks (*p* < 0.001), HC neurons at 8 weeks (*p* < 0.001), and BD neurons at 8 weeks (*p* = 0.007). **D** Peak fluorescence obtained per cell. Significant two-way ANOVA (F_7,2966_ = 66.4, *p* < 0.001) demonstrates that HC-NPCs had a higher peak fluorescence than BD-NPCs (*p* < 0.001), HC neurons differentiated for 2 weeks (*p* < 0.001), BD neurons at 2 weeks (*p* < 0.001), HC neurons at 8 weeks (*p* < 0.001), and BD neurons at 8 weeks (*p* < 0.001). BD-NPCs had significantly higher peak fluorescence than BD neurons at 2 weeks (*p* < 0.001), and HC neurons at 8 weeks (*p* < 0.001). HC neurons at 2 weeks had significantly higher peak fluorescence than BD neurons at 2 weeks (*p* = 0.001) and HC neurons at 8 weeks (*p* < 0.001). **E** Ca^2+^ re-addition assay after treatment with the ORAI channel blocker YM58483 compared to influx from (**C**). Data is presented as mean (red and blue curves) ± s.e.m (grey ribbon). **F** AUC analysis was conducted using the ANOVA described in (**C**) and demonstrates no significant difference between HC- and BD-NPCs (*p* = 0.20). **G** Peak fluorescence analysis was conducted using the ANOVA described in (**D**) and demonstrates no significant difference between HC- and BD-NPCs (*p* = 0.06). ^*^p < 0.05, ^**^p < 0.01, ^***^p < 0.001; ANOVA with Tukey’s post hoc test when appropriate. Each of the 6 cell lines are represented in the Ca^2+^ imaging where *n* > 198 cells per line in NPCs and *n* = 100 per line for differentiated neurons.

Some of the most reproducible cellular results concerning BD are those relating to Ca^2+^ signaling [16], a crucial component of central nervous system development and various aspects of neuroplasticity [18–20]. Apart from specialized voltage-gated Ca^2+^ channels and glutamatergic receptors, neurons also employ store-operated Ca^2+^ entry (SOCE [21, 22]; Fig. 1A). Recent evidence found that resting neurons have the most active SOCE [23], which functions as a key mediator of cell survivability [24] and neural differentiation [25]. Given the influence SOCE exerts on neurodevelopment and cellular excitability, we formulated the idea that aberrant activity could play a part in BD neuropathology.

Here, we used 6 iPSC-derived NPC lines from 3 patients with type I BD (BD-NPCs) and 3 healthy controls (HC-NPCs) to study SOCE activity at the progenitor and differentiated neuron stages. Our approach allowed us to uncover a difference in the structure and function of BD-NPCs and neurons differentiated for 2 weeks compared to the HC-NPCs and neurons, namely a reduction of SOCE-dependent Ca^2+^ influx. Due to the range of processes affected by Ca^2+^ dynamics, we chose to employ RNA-sequencing (RNA-seq) and found that HC- and BD-NPCs contained a distinct transcriptome including increases in genes regulating neuronal differentiation. Next, functional assays revealed a consistent tendency towards differentiation due to decreased proliferation and increased neurite outgrowth, and characterization of iPSC-derived cerebral organoids revealed marked differences in 3D spatial patterning between HC and BD specimens. Finally, we measured an increase in the expression of the *let-7* family of microRNAs (miRNAs) in BD-NPCs and of miR-34a in BD neurons—a result that is consistent with previous observations [26, 27]. Together, our results showcase a developmental component to BD that may be the key to uncovering new etiological factors responsible for the disease.

## Material and methods

### iPSC reprogramming, NPC derivation, and differentiation to cortical glutamatergic projection neurons

Human fibroblasts from healthy control and BD subjects enrolled in research studies at the Massachusetts General Hospital (Department of Psychiatry, Center for Genomic Medicine) were grown from dermal skin punches and reprogrammed as previously described [28].

When differentiating as 2D monolayers and neurospheres, cells were cultured in media containing 69.25% DMEM (Gibco), 29.25% Ham’s F12 (Gibco), 2% B27 (Gibco), 50 units/mL penicillin and 50 µL/mL streptomycin (Gibco) with a cortical glutamatergic projection neuron-like track, as characterized previously (29). Once in differentiation media, the cells were maintained without passage and media was changed every 2-3 days. Use of the patient-derived iPSC lines and generation of cerebral organoids for this study was performed at the University of Guelph and was approved by the University of Guelph Research Ethics Board (REB 17-11-012). All methods were carried out in accordance with relevant guidelines and regulations.

Please see Supplemental Information for detail about Cell culture, Ca^2+^ imaging, Puncta formation assay, Seahorse mitochondrial stress test, EdU assay, TUNEL assay, Neurosphere assay, Immunocytochemistry, Immunoblotting, RNA-seq, Cerebral organoid generation and staining, NanoString miRNA profiling, Real-time quantitative PCR, and Statistics.

## Results

### BD-NPCs and neurons have attenuated SOCE-specific Ca^2+^ influx

Most research to date about neuronal Ca^2+^ dynamics in relation to BD focuses on glutamatergic receptors and voltage-gated channels in differentiated neurons, whereas little attention has been directed towards possible Ca^2+^ mobilization differences at the NPC stage via SOCE. So, our first goal was to assess Ca^2+^ influx from SOCE in HC- and BD-NPCs, neurons differentiated for 2 weeks, and neurons developed to synaptic maturity at 8 weeks (Fig. S1; [29]). Using the Ca^2+^ indicator Fluo-4, Ca^2+^ imaging was conducted in NPCs, 2-week immature neurons, and 8-week mature neurons under a cocktail of inhibitors designed to abolish the activity of alternative Ca^2+^ pathways, namely NMDARs, AMPARs, and L-type voltage-gated Ca^2+^ channels [23]. In the presence of the sarcoendoplasmic reticulum Ca^2+^ transport ATPase (SERCA) pump inhibitor thapsigargin (Tg, a potent activator of SOCE), and after a brief incubation in Ca^2+^-free media, the cells were exposed to 2 mM Ca^2+^ and the relative increase in fluorescence from baseline (an average of fluorescence between *t* = 0 s and *t* = 180 s) was recorded (Fig. 1B; [30]). Curves are shown as a single average for all HC-NPCs or neurons from 50–100 cells in each of the 3 lines (red) and a separate, similarly obtained average for BD-NPCs or neurons. Area-under-the-curve (AUC; Fig. 1C) analysis from *t* = 185 s to *t* = 600 s and peak fluorescence analysis (Fig. 1D) revealed two key results. First, the AUC of HC-NPCs was 1.2x bigger, and the peak fluorescence 1.3x higher than BD-NPCs *(p* < 0.001), suggesting an attenuation in SOCE at this stage of neural differentiation. This was confirmed ratiometrically with Fura-2, which also showed that this disparity is not due to a difference in baseline Ca^2+^ concentrations between HC- and BD-NPCs (Fig. S2). Interestingly, at the 2-week timepoint this disparity was reduced to 1.1x for the AUC and 1.2x for the peak fluorescence measures, but still significant (*p* < 0.001). Finally, at the 8-week timepoint neither AUC nor peak fluorescence were statistically different between HC and BD neurons (*p* = 0.22). Importantly, significance was achieved even with inter- and intra-patient variability, highlighting the strength of this trend (Fig. S2).

To determine if these differences in Ca^2+^ influx were due to a dysregulation in SOCE, we repeated this experiment in the NPCs under SOCE-inhibiting conditions with the ORAI channel blocker YM58483 (Fig. 1E; [31]). As expected, the disparity in AUC (Fig. 1F) and peak fluorescence (Fig. 1G) between HC- and BD-NPCs was reduced and not statistically significant (*p* = 0.20 and *p* = 0.06, respectively) in the presence of YM58483. Interestingly, though, a small increase in Fluo-4 fluorescence remained following Tg application in both the BD-NPCs and the YM58483-treated HC-NPCs. We believe that these residual increases in SOCE are due to incomplete suppression since it has been previously demonstrated that germline deletion of SOCE effectors is more efficient in abolishing SOCE signal than pharmacological inhibition [32]. Of note, this residual increase could also result from the activity of Transient Receptor Potential Canonical (TRPC) channels since this pathway has been shown to influence SOCE activity in a number of contexts, including promoting the switch from proliferation to differentiation in A2B5^+^ NPCs [33, 34]. Therefore, to ensure that the influx we measured here is ORAI-dependent, and that the residual increase is not caused by other key Ca^2+^ channels, we inhibited TRPC4/5 and the activity-dependent Ca^2+^ channel NDMAR in succession in a representative HC and BD line to assess any effects those other players may have on the disparity between HC- and BD-NPCs (Fig. S3). Interestingly, difference in Ca^2+^ influx persisted *(p* < 0.001) through all our inhibition attempts. Altogether, these Ca^2+^ imaging results support the hypothesis that SOCE induced by Tg application is dysregulated in BD-NPCs and persists through the 2-week stage of differentiation but is resolved by the time the neurons reach 8-week differentiation.

### BD-NPCs have decreased STIM2 puncta formation during SOCE activation

**Fig. 2:**
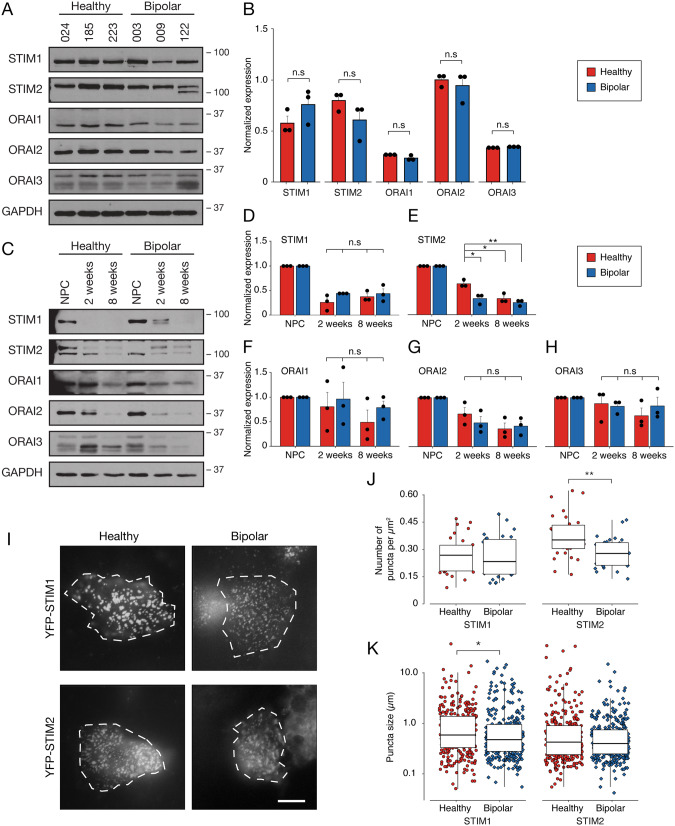
SOCE effector expression and puncta quantification are unique between HC- and BD-NPCs and neurons. **A** SOCE effector immunoblotting on all 6 cell lines at the NPC stage with GAPDH used as a loading control. **B** Subsequent quantification of the western blot shows no significant differences in the expression of any effector (STIM1: t_4_ = 1.5, *p* = 0.21; STIM2: t_4_ = 1.63, *p* = 0.18; ORAI1: t_4_ = 1.96, *p* = 0.12; ORAI2: W = 5, *p* = 1; ORAI3: t_4_ = 1.0, *p* = 0.37, *n* = 3 for all effectors). **C** SOCE effector immunoblotting using representative cell lines at the NPC, 2-week, and 8-week stage of differentiation. Intensity was divided by intensity of GAPDH for normalization. Quantification was done for all 5 SOCE effectors using all 6 lines (*n* = 3 per condition with 1 replicate per cell line) and normalization was done by dividing protein band intensity by intensity of GAPDH. No interaction effect was observed for STIM1 (**D**; F_3,8_ = 1.35, *p* = 0.32), ORAI1, (**F**; F_3,8_ = 0.56, *p* = 0.66), ORAI2 (**G**; F_3,8_ = 1.19, *p* = 0.37), or ORAI3 (**H**; F_3,8_ = 0.49, *p* = 0.70). A significant interaction was observed for STIM2 (**E**; F_3,8_ = 11.47, *p* = 0.0028) showing a difference between HC and BD neurons at 2 weeks (*p* = 0.012), HC neurons at 2 weeks and BD neurons at 8 weeks (*p* = 0.0027), and HC neurons at 2 weeks and HC neurons at 8 weeks (*p* = 0.012). **I** All 3 cell lines from each condition (healthy, left; BD, right) were transiently transfected with YPF-STIM and imaged for YFP-STIM1 (top) and YFP-STIM2 (bottom) puncta formation 10 mins after the addition of 2 µM Tg. Cell bodies are outlined with a dotted white line. Scale bar represents 10 µm. **J** Subsequent quantification shows significantly more YFP-STIM2 puncta formation in HC-NPCs than BD counterparts (W = 667, *p* = 0.0011, *n* = 30 per condition, 10 per cell line). **K** Quantification of puncta size revealed a significant increase for HC-NPCs with YFP-STIM1 (W = 49328, *p* = 0.042, *n* = 300 per condition, 100 per cell line, 10 per cell). Data is presented as mean±s.e.m. n.s. signifies *p* > 0.05, ^*^p < 0.05, ^**^p < 0.01; Student’s *t*-test or ANOVA with Tukey’s HSD post hoc test was used for parametric data, Mann Whitney U test was used for non-parametric data.

Next, we aimed to test whether the attenuation of SOCE measured in the BD-NPCs could be attributable to differences in the abundance of core effectors (STIM1/2, ORAI1/2/3). As shown in Fig. 2A, immunoblot analysis for STIMs and ORAIs revealed no apparent difference between HC- and BD-NPCs for all tested proteins. This was confirmed by quantification of the immunoblot results showing that intensity for all SOCE effectors at the NPC stage were not statistically significant (Fig. 2B), namely STIM1 (*p* = 0.21), STIM2 (*p* = 0.18), ORAI1 (*p* = 0.12), ORAI2 (*p* = 1), and ORAI3 (*p* = 0.37). In light of this result, we conclude that a different factor, such as membrane lipid composition [35] or physiological activation of ORAI channels [36], must be responsible for the attenuated Ca^2+^ influx at this stage of differentiation. It is interesting to note here that there is a qualitative, though statistically insignificant, decrease in STIM1 for BD lines 009 and 122. These are lithium-insensitive lines where 003 is lithium sensitive, and so future research with high sample sizes should consider differences in SOCE effector expression between lithium-sensitive and lithium-insensitive patient-derived cell lines. Additionally, a double band can be observed in STIM2 for patient line 122 and STIM2 is known to have several isoforms that differ slightly in weight. STIM2.2 is the canonical protein that can exist at 2 different sizes: a larger, more phosphorylated protein at 115 kDa and a smaller, less phosphorylated protein at 105 kDa [37]. Additionally, STIM2.1 and STIM2.3 have been discussed in the literature. STIM2.1 has found to be a larger, SOCE-inhibiting isoform while STIM2.3 is smaller, but little is known about its function [38]. In this case, we speculate that this band is the STIM2 isoform, STIM2.3.

We also immunoblotted SOCE effectors over the course of differentiation (Fig. 2C–H). While there were no significant differences in the expression of STIM1 (*p* = 0.32), ORAI1 (*p* = 0.66), ORAI2 (*p* = 0.37), or ORAI3 (*p* = 0.70), we found that HC neurons differentiated for 2 weeks had significantly higher expression of STIM2 than BD neurons after 2 weeks of differentiation (*p* = 0.012) and after 8 weeks of differentiation (*p* = 0.012). Importantly, 2 bands were present around the molecular weight of STIM2, which we suspect to be the differentially phosphorylated versions (39). The differences in expression presented here are concerning the less phosphorylated, smaller STIM2 protein. This suggests that the SOCE attenuation in the BD neurons over differentiation may be attributed, at least in part, to a relative decrease in the expression of STIM2.

To initiate SOCE, STIM proteins move to the plasma membrane and interact with ORAI channels to open them [39]. This event can be visualized by overexpressing STIM tagged to a fluorescent protein and monitoring the formation of discrete puncta [23, 40]. Observing a lower number of STIM puncta or smaller formations would support our finding of reduced Ca^2+^ influx through SOCE. To test this, we transfected our NPC lines with YFP-tagged STIM1 or YFP-tagged STIM2 (Fig. 2I) and treated the cells with 2 µM Tg for 10 min. Live NPCs from each group were then imaged and YFP-STIM puncta counted and measured. For YFP-STIM1, no difference between HC- and BD-NPCs was found for the number of puncta formed (*p* = 0.89; Fig. 2J); however, we observed that puncta size in HC-NPCs was on average 0.08 µm larger (*p* = 0.042; Fig. 2K). For YFP-STIM2, we counted 0.75x less puncta on BD-NPC membranes than HC (*p* = 0.0011; Fig. 2J), but no difference in size of puncta between the two groups. Importantly, it has been observed that STIM puncta can be static and, therefore, persistent even in the absence of store depletion [41]. In our NPCs, however, these static puncta were too sparse to quantify (Fig. S4). Based on those results, we propose that the lower Ca^2+^ influx from SOCE in BD-NPCs is primarily due to a decreased ability to form puncta on the cell surface in the NPCs.

### Mitochondria function normally between HC- and BD-NPCs

Mitochondria play a key role in sequestering and shaping cytosolic Ca^2+^ rises [42], and mitochondrial dysfunction has been previously implicated as a contributing factor to BD pathophysiology [43]. Moreover, there is continuous Ca^2+^ exchange between ER and mitochondria [44]. Therefore, to ensure that mitochondria biology is consistent between the HC- and BD-NPCs, we assessed this fundamental cellular process using the Seahorse Mito Stress Test. No significant differences were observed between HC- and BD-NPCs for any parameters measured by the (Fig. S5): non-mitochondrial respiration (*p* = 0.4206), basal respiration (*p* = 0.6041), maximal respiration (*p* = 0.2201), ATP production (*p* = 0.4570), spare respiratory capacity (*p* = 0.3854) and coupling efficiency (*p* = 0.4348). This analysis shows that the differing Ca^2+^ dynamics that we found in our BD-NPC lines cannot be attributed to mitochondrial dysfunction.

### Gene expression profiles are unique between HC- and BD-NPCs

**Fig. 3:**
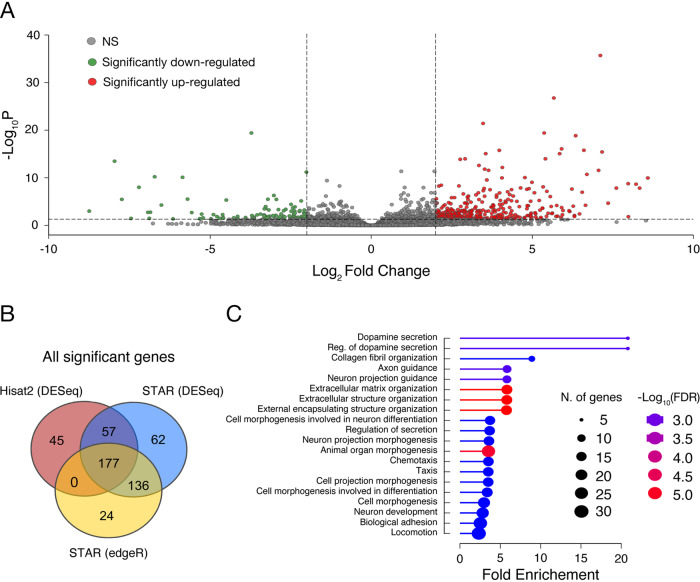
RNA-seq demonstrates a distinct transcriptome between HC- and BD-NPCs. **A** Volcano plot of the fold change of all differentially expressed genes in BD-NPCs including those with a log_2_ fold change significantly greater (red) or less than (green) 2 (*p* < 0.05) using STAR-DESeq2. 283 genes were found to be upregulated and 77 were downregulated. **B** To avoid bias from the analysis software, we re-quantified DEGs using 2 other methods, Hisat2-DESeq2 and STAR-edgeR. The Venn diagram represents the common DEGs found using each technique. **C** Gene Ontology analysis of the common DEGs ordered by category fold enrichment (ratio of the number of genes over/underexpressed compared to total number of genes associated with that biological function). Circle size illustrates number of DEGs involved in each function. Color demonstrates false discovery rate (FDR) from -log_10_3.0 (blue) to - log_10_5.0 (red).

Given the finding that SOCE is dysregulated in the BD-NPCs, our next goal was to track what cellular differences this dysregulation yields. To first pinpoint transcriptional differences in a non-biased and high-throughput manner, we employed RNA-seq on the 6 iPSC-derived NPC lines using a multi-procedural approach. From STAR-DESeq2, we discovered that BD-NPCs had 283 upregulated genes and 77 downregulated genes with a log_2_ fold change ≥2 or ≤ -2 (*p* < 0.05) compared to HC-NPCs (Fig. 3A, Supplementary File 1). Of those, the top 20 most differentially expressed include the upregulated genes *CFI*, *TGFBI*, and *CXCL12*, and the downregulated gene *TBX1*. As any single method of differentially expressed gene (DEG) quantification has bias, we re-conducted the analysis with Hisat2-DESeq2 and STAR-edgeR. Commonly between the 3 analysis methods there were 148 upregulated and 29 downregulated genes (Fig. 3B). Gene Ontology (GO) analysis from *geneontology.org* powered by the PANTHER classification system from revealed that the top enriched biological processes from the common upregulated genes include processes implicated in neurodevelopment, such as “neuron projection guidance”, “extracellular matrix organization”, “cell morphogenesis involved in neuron differentiation”, and “neuron development”. Other enriched processes include “regulation of secretion”, “chemotaxis”, “biological adhesion”, and “locomotion” (Fig. 3C). Taken together, these results suggest that attenuation of SOCE in BD-NPCs could be responsible for wide-scale changes in gene expression affecting neurodevelopmental processes such as neurite extension, migration, and differentiation.

### BD-NPCs exhibit lower rates of proliferation, increased neurite outgrowth, and smaller neurosphere formation

**Fig. 4:**
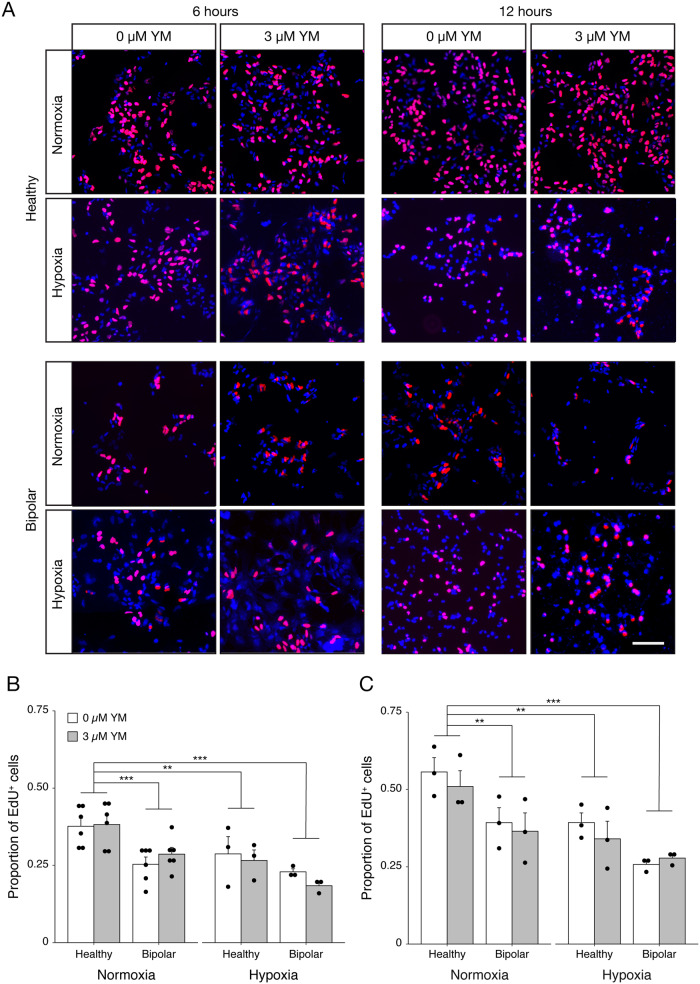
EdU assay demonstrates lower rates of proliferation and higher resilience to stress in BD-NPCs. **A** Representative immunofluorescent images of HC- and BD-NPCs without and with SOCE inhibition via 3 µM YM58483 (YM) after 6 and 12 h of incubation under normoxic and hypoxic conditions with the thymidine analog EdU (red). DAPI is shown in blue. Scale bar represents 100 µm. **B** Quantification of EdU^+^ cells / DAPI after 6 h of incubation under normoxia and hypoxia to measure proliferation rate. There was a significant interaction between condition and oxygen level (F_3,32_ = 13.7, *p* = 6.3 × 10^−^^6^, *n* = 12 per condition, 4 per cell line) that demonstrates a higher rate of proliferation in HC-NPCs in normoxia compared to BD-NPCs in normoxia (*p* = 0.00039), HC-NPCs in hypoxia (*p* = 0.0072), and BD-NPCs in hypoxia (*p* = 8.6 × 10^-6^). **C** These results were consistent after 12 h in the different oxygen conditions: a significant interaction effect (F_3,20_ = 14.4, *p* = 3.1 × 10^-5^, *n* = 6 per condition, 2 per cell line) demonstrates a higher rate of proliferation in the HC-NPCs in normoxia compared to BD-NPCs in normoxia (*p* = 0.0059), HC-NPCs in hypoxia (*p* = 0.0030), and BD-NPCs in hypoxia (*p* = 1.37 ×10^-5^). Model testing was used to determine a lack of evidence to include YM58483 treatment to the model, and so results at 0 µM and 3 µM YM treatment were pooled for both the 6- and 12-hour analyses. Data is presented as mean ± s.e.m. ^**^p < 0.01, ^***^p < 0.001; ANOVA with Tukey’s post hoc test when appropriate.

Our RNA-seq data hints at developmental differences between HC- and BD-NPCs. This, along with SOCE contributing to proliferation and differentiation [25], led us to conduct experiments to compare neurodevelopmental phenotypes between our two conditions. To assay proliferation, HC- and BD-NPCs were incubated with the thymidine analog 5-ethynyl-2’-deoxyuridine (EdU) for 6 and 12 h with and without YM58483 inhibition. To test how the NPCs responded to environmental stress, we compared cells under normoxic and hypoxic (O_2_ = 1%) conditions (Fig. 4). Due to a lack of significance between the untreated and treated groups, 0 µM and 3 µM YM58483 data points were pooled together for analysis. At both 6 and 12 h, HC-NPCs in normoxia had the fastest rate of proliferation compared to BD-NPCs in normoxia (*p* < 0.001 and *p* = 0.003), HC-NPCs in hypoxia (*p* = 0.007 and *p* = 0.006), and BD-NPCs in hypoxia (*p* < 0.001). The foremost conclusion from this data is that BD-NPCs have a slower rate of proliferation than their healthy counterparts, though this difference cannot be attributed to SOCE dysregulation. Of note, this significance is abolished in hypoxic conditions, meaning both HC- and BD-NPCs have a similar rate of proliferation under low levels of O_2_. This suggests a higher tolerance to stress in BD-NPCs compared to healthy counterparts, an interesting and unexpected finding. Alternatively, BD-NPCs already have a low rate of proliferation in normoxia and so any further reduction may be difficult to detect. Instead, oxidative stress present in BD-NPCs under basal conditions may be the source of this reduction in proliferation [45]. To ensure that this difference in proliferation was not due to increased cell death in the BD-NPCs, a TUNEL assay was conducted under normoxia and hypoxia between our 2 conditions (Fig. S6). There were no significant differences between cell death in any treatment group.

**Fig. 5:**
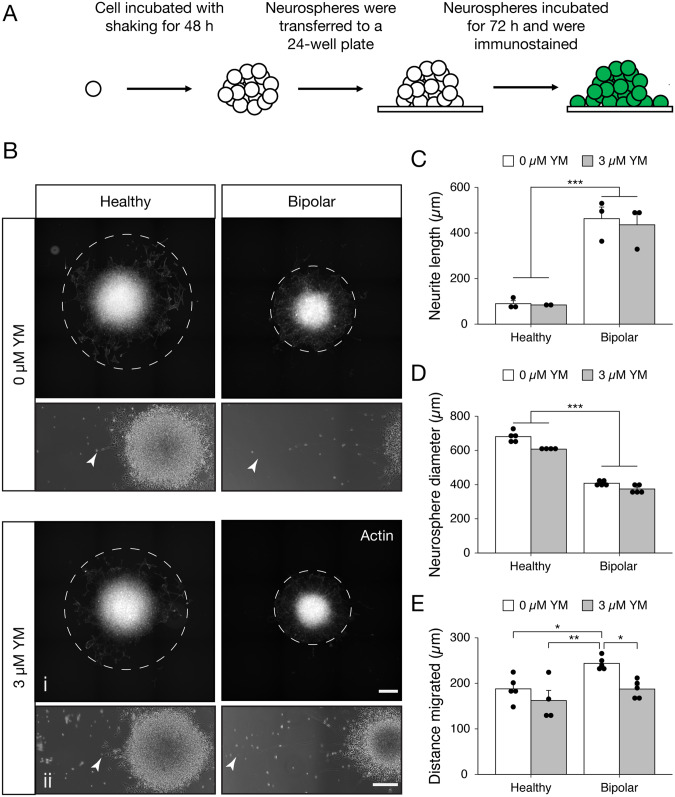
Neurosphere assay uncovers longer neurites, farther migration, and smaller sized spheres in BD-NPCs. **A** A schematic depicting the methodology of the assay. **B** Representative fluorescent (i) and phase contrast (ii) micrographs for actin staining and neurite extension of HC and BD- derived neurospheres without (top) and with (bottom) SOCE inhibition (0 µM or 3 µM YM58483 (YM), respectively). Dotted lines migration of NPCs from the neurospheres. Arrow heads highlight the tip of the longest neurite imaged. Scale bars represent 200 µm. **C** Average length of neurites. 0 µM and 3 µM YM were pooled due to a lack of significance (W = 18, *p* = 1, *n* = 3), after which BD neurospheres had significantly longer extensions (W = 36, *p* = 0.002, *n* = 6). **D** Average size (diameter) of neurospheres measured. 0 µM and 3 µM YM data were pooled due to insignificance (W = 72, *p* = 0.11, *n* = 5), after which BD neurospheres were significantly smaller (W = 0, *p* = 1.1 ×10^-5^, *n* = 10). **E** Average distance cell bodies migrated from the neurosphere. Significant interaction (F_3,15_ = 7.21, *p* = 0.0032) demonstrates farther migration in untreated BD neurospheres (*n* = 5) compared to untreated HC (*p* = 0.028; *n* = 5), treated HC (*p* = 0.0027, *n* = 4) and treated BD- (*p* = 0.027, *n* = 5) derived neurospheres. Due to inadequate neurosphere formation with the other cell lines, data used only healthy line PSC-01-223 and BD line PSC-01-003 and is presented as mean ± s.e.m. ^*^p < 0.05, ^**^p < 0.01, ^***^p < 0.001; Mann–Whitney *U* test.

To assay migration and differentiation capacity, we employed a neurosphere assay in a representative HC- and BD-NPC line with and without pharmacological SOCE inhibition. HC and BD neurospheres were generated and plated onto Geltrex-coated plates. After a 72 h incubation period in differentiation media [29], the neurospheres were fixed and stained for visualization (Fig. 5A, B) and quantification in Image J (Fig. 5C–E). A striking result was uncovered in the amount of neurite outgrowth in the BD neurospheres. The distinction between HC and BD neurospheres is apparent both qualitatively (Fig. 5B) and quantitatively (Fig. 5C–E). Neurites developed from BD neurospheres were on average 362 nm longer than HC counterparts, regardless of YM58483 treatment condition (*p* = 0.002). An additional observation was the size of the neurospheres, a parameter that has been linked to the self-renewal capacity of NPCs [25]. BD neurospheres were 255 nm smaller in diameter than HC neurospheres, again regardless of YM58483 treatment condition (*p* < 0.001; Fig. 5D). In terms of migration, untreated BD-NPCs migrated 55.8 nm farther than untreated HC-NPCs on average, and 81.6 nm farther than treated HC-NPCs (*p* = 0.028 and *p* = 0.0027, respectively). Interestingly, they also migrated 56.4 nm farther than YM58483-treated BD-NPCs (*p* = 0.027; Fig. 5E). Taken together, these data suggest a tendency towards differentiation in the BD-NPCs.

### Cerebral organoid model reveals differences in subventricular zone (SVZ) formation between HC and BD organoids

**Fig. 6:**
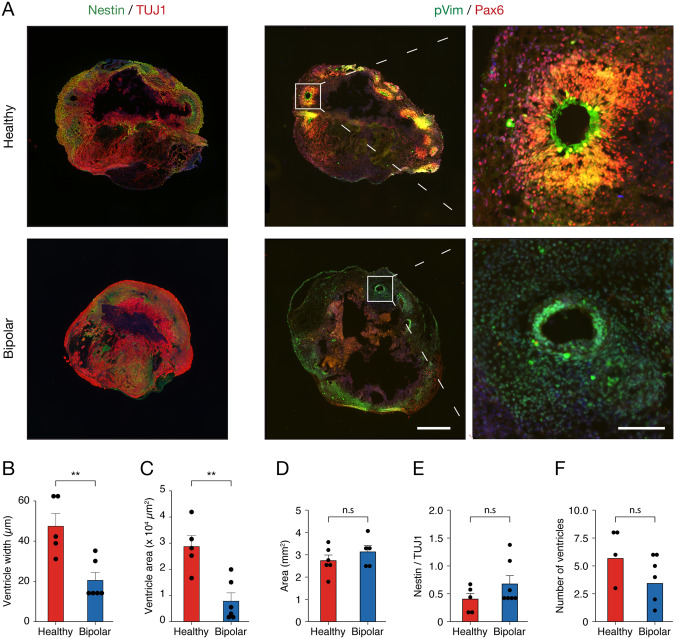
BD organoids have smaller and thinner SVZs compared to HC organoids. **A** Micrographs of a representative HC and BD organoid matured to 50 days. Organoids were co-stained for radial glia marker Nestin and immature neuron marker TUJ1 or for migration marker pVim and proliferation marker PAX6. Inlet shows an example SVZ. Scale bars represent 500 µm and 100 µm. **B**, **C** Average ventricular width and area per organoid. There was a significant decrease in width (t_9_ = 3.74, *p* = 0.0046) and area (t_9_ = 4.07, *p* = 0.0028) in the BD organoids (*n* = 3, healthy: *n* = 6). At least 1 organoid per cell line was used in each analysis. Data is presented as mean ± s.e.m; ^**^p < 0.01, n.s signifies *p* > 0.05; Student’s *t*-test. **D** Average area of organoids. No significant differences were found between HC (*n* = 6) and BD (*n* = 4) organoids (t_8_ = 1.05, *p* = 0.32). **E** Ratio of Nestin/TUJ1 used to measure organoid maturity. Compared to HC (*n* = 5), BD (*n* = 4) organoids show no difference in relative Nestin expression (W = 22, *p* = 0.53). **F** Average number of ventricles per organoid. No significant differences were observed in BD- (*n* = 4) compared to HC (*n* = 6) organoids (t_11_ = 1.35, *p* = 0.20).

To further support the differences found between the HC and BD conditions above, we prepared 3D cerebral organoids with iPSCs from the same individuals as our NPC lines to characterize SVZ regions. Specifically, we focused on overall differentiation and size at the 50-day maturation timepoint (Fig. 6A). Comparison between the HC and BD groups revealed that SVZs were 2.3x thinner (*p* = 0.0046; Fig. 6B) and occupied 3.7x smaller area (*p* = 0.0028; Fig. 6C) in BD cerebral organoids. As SVZs are the sites of ongoing neurogenesis in a developing and adult brain, this result suggests that development in BD brains may be affected. Area (Fig. 6D), relative expression of Nestin (Fig. 6E), and number of ventricular zones (Fig. 6F) were not significantly different. In addition, a set of HC cerebral organoids was also treated with 3 µM and 6 µM YM to test SOCE-specific processes in this model, though no differences were observed between untreated and YM58483-treated organoids for number of ventricles, width of the SVZ, or subventricular area (Fig. S7). This result is not surprising, in our opinion, considering that deep pharmacological penetration into organoid tissue is difficult to achieve [46]. Overall, these results strengthen the idea that aspects of neurodevelopment are dysregulated in BD by introducing SVZ morphology as a specific phenotypic difference.

### *let-7* family and miR-34a are differentially expressed in BD-NPCs and neurons

**Fig. 7:**
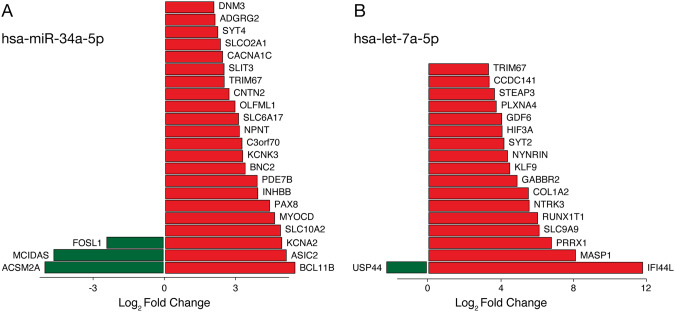
Cross referencing predicted targets of has-miR-34a-5p and *hsa-let-7a-5p* with DEGs from RNA-seq between HC- and BD-NPCs. **A** miR-34a was shown to be upregulated in BD neurons at 4 and 6 weeks of differentiation in a NanoString analysis, so its predicted targets were compared to DEGs from the RNA-seq presented earlier. The plot shows putative targets and their respective expression levels. **B** *hsa-let-7a* and its family members with similar putative targets were shown to be upregulated at the NPC stage in BD lines in a NanoString analysis, so predicted targets of *hsa-let-7a-5p* were compared to DEGs from the RNA-seq presented earlier. The plot shows putative targets and their respective expression levels. Green represents downregulated genes while red represents upregulated genes.

Finally, miRNAs are linked to numerous cellular processes, including neurodevelopment, and have been implicated as biomarkers for neuropsychiatric conditions like schizophrenia and major depression [47, 48]. Consequently, we sought to probe for differences in miRNA expression between our HC- and BD-NPC lines in addition to two others, GM08330 and GM05440, as described previously by Bavamian and colleagues [26]. Of special interest, previous studies found that miR-34a overexpression is associated with significant reduction of STIMATE (also known as TMEM110), which is a crucial protein in SOCE regulation [26, 49]. Moreover, miR-34a is also known to influence proliferation, morphogenesis, and differentiation of newborn neurons [50, 51]. Using Nanostring miRNA profiling of our NPC lines, we found higher counts of miR-34a at 4 weeks (3.24x higher) and 6 weeks (2.4x higher) of neuronal differentiation in BD lines, but not in NPCs or 2-week differentiated neurons (Supplementary File 2). This was further confirmed in our cell lines using qPCR (Fig. S8). When comparing the targets of this miRNA to the DEGs from our STAR-DESeq2 RNA-seq analysis, we identified 22 upregulated and 3 downregulated genes considered to be target of this miRNA (Fig. 7A). Of note, the downregulated gene *FOSL1* is part of the FOS family, which are genes canonically known to influence proliferation and differentiation in neurons through their dimerization with JUN proteins [52]. Above all, our results concerning miR-34a support previous data from Bavamian and colleagues that used a different BD patient-derived NPC line (5440). While our findings suggest accelerated differentiation, they claim that this overexpression stunts neuronal morphology and synaptogenesis [26]. Further research is needed to consolidate these converse results.

Also of interest, the entire family of *let-7* was upregulated in BD-NPCs including *7a* (76x higher), *b* (8.2x higher), *c* (10x higher), *d* (30x higher), *e* (5.7x higher), *f* (15x higher), *g* (18x higher), and *i* (25x higher; Supplementary File 2). Since the predicted targets of all these family members are highly similar, we compared the targets of *let-7a-5p* to the DEGs in our RNA-seq analysis and found 17 upregulated and 1 downregulated gene in common (Fig. 7B). Ubiquitin-specific protease 44 (*USP44*) regulates the spindle assembly checkpoint and has been implicated in intellectual disability, but never BD [53]. Future research is needed to establish its role in BD.

While this comparison between the RNA-seq and Nanostring analysis demonstrates that DEGs may be due to translational regulation by miRNAs, a more comprehensive analysis on non-coding RNAs must be done to fully understand this process. Firstly, alternative polyadenylation alters miRNA binding capabilities to the 3’UTR of transcripts [54]. Additionally, crosstalk between miRNAs and other non-coding RNAs contribute to translational regulation more than miRNAs alone [55]. Finally, miRNAs have been found to increase translational efficiency in addition to inhibiting it [56]. Overall, processes of differentiation rely on careful manipulation of gene expression and dysregulation in these mechanisms contribute greatly to pathology of disease. Here we uncovered a new potential mechanism of dysregulation through differential expression of miRNAs crucial for neurodevelopment.

## Discussion

BD is a complex disorder for which various theories about its etiology, like mitochondrial dysfunction (reviewed in [45]) and inositol depletion (reviewed in [57]), have been proposed. None of the models presented to date, however, have been able to adequately explain the pathology nor the cause of the disease. Alternative efforts in recent years have been to examine the role of neurodevelopment in NPCs, with much of that research suggesting that aberrant Ca^2+^ dynamics play a central part [15, 16]. The role SOCE plays in these aberrant Ca^2+^ dynamics has been explored in non-neuronal surrogate cell models of BD such as lymphoblastoid cells [58, 59], but not NPCs or neuronal models of the condition. Furthermore, few studies have examined the contribution of individual SOCE effectors and more often focused on a single isoform, in particular STIM1 and ORAI1. Here, our goal was to explore SOCE-related developmental defects in BD-NPCs in a more holistic manner and our conclusions are summarized in Table 1.

**Table 1:** Summary of results.

Experiment	Outcome
Ca^2+^ imaging	BD-NPCs have reduced Ca^2+^ influx from SOCE.
Effector quantification	NPCs have no differences in effector expression. Immature neurons have reduced STIM2 expression.
Puncta formation assay	BD-NPCs form smaller STIM1 puncta and less STIM2 puncta.
RNA-sequencing	BD-NPCs have a distinct transcriptome with enriched neurodevelopmental processes.
Proliferation assay	BD-NPCs have slower rates of proliferation.
Neurosphere generation	BD-NPCs form smaller neurospheres, have more migration, and longer neurite extension.
Organoid generation	BD organoids have subventricular zones with smaller width and area.
Nanostring miRNA analysis	BD-NPCs have increased expression of the *let-7* miRNA family and BD immature neurons have increased expression of miR-34a.

Our Ca^2+^ imaging analysis revealed that SOCE was indeed dysregulated in BD-NPCs as Ca^2+^ influx via this pathway was barely measurable in response to Tg application (Fig. 1). Further supporting this claim, pre-treatment with the ORAI channel blocker YM58483 significantly reduced the SOCE signal in the HC-NPCs but not the BD-NPCs (Fig. 1). From this, we conclude that the BD-NPCs have minimal SOCE activity. Importantly, this disparity persists at 2 weeks of differentiation but is abolished after 8 weeks, when the neurons reach maturity (Fig. 1). This decrease in activity over differentiation was expected since previous studies have shown that SOCE promotes self-renewal in progenitors, and the decrease of this activity coincides with differentiation [25, 32, 60]. Furthermore, as the neurons begin to express more specialized Ca^2+^ channels, SOCE becomes less vital for Ca^2+^ influx. When examining protein expression, the effectors also showed an overall decrease over differentiation. Of note, STIM2 protein expression was significantly decreased in a pattern that exactly matched that of the differences in Ca^2+^ influx. STIM2 in BD neurons was consistent with that of HC neurons at an early stage of differentiation, highlighting a premature differentiation phenotype dependent on its expression. Following this discovery in the neurons, we sought to understand why a disparity in Ca^2+^ influx existed between HC- and BD-NPCs. Immunoblot analysis of SOCE effectors revealed no differences between the 2 conditions at the NPC stage (Fig. 2). Since another cause for decreased influx could be due to aberrant puncta formation, we tested this scenario with transiently transfecting YFP-STIM1/2 constructs and found that the BD-NPCs could not form as many STIM2-specific puncta. This is a finding that may have relevant implications for the pathogenesis and progression of BD, especially with respect to STIM2. Specifically, of the two effectors, STIM2 is more widely expressed and is the most prominent effector of SOCE in the mouse hippocampus [61]. A key feature of STIM2 is its role in regulating basal Ca^2+^ concentrations in the cytosol due to its higher dissociation constant (K_D_) and sensitivity to smaller Ca^2+^ fluctuations [41, 62–64]. While baseline Ca^2+^ concentrations could not be measured in this study due to the non-ratiometric dye used, it would be of interest to examine differences in these levels over differentiation given that mature neurons from BD and schizophrenic patients have increased basal cytosolic Ca^2+^ concentrations [65]. Further, while STIM1 has been shown to be pivotal for self-renewal and proliferation [25], the specific effects of STIM2 on differentiation have not been explored.

Since intact mitochondria are required for proper SOCE function, we ensured that the resulting phenotypes observed are due to SOCE dysfunction and not mitochondrial effect [44]. Upon completion of a Seahorse Mito Stress Test, we have concluded that the HC- and BD-NPCs have similar mitochondrial function and no defects (Fig. S5), which is in line with previous studies using IPSC-derived HC- and BD-NPCs [66]. While this may seem inconsistent with other findings using hippocampal DG granule cell-like neurons derived from BD patients showing mitochondrial deficits [67], we would like to note the changes that are occurring in mitochondria during neurogenesis—including mitochondria morphology, RNA expression profiles, and metabolic shift from glycolysis to OXPHOS [68]—may account for these differences. Further research comparing mitochondrial functionality on different stages of neurogenesis is needed to explain this phenomenon.

Next, we looked for possible differences in functionality of the BD-NPCs to explain the tendency observed in the RNA-seq towards differentiation. For this aim we used EdU proliferation assay, in which BD-NPCs exhibited a significantly reduced rate of proliferation compared to the HC-NPCs (Fig. 4). To take it one step forward, we also tested the proliferation under a hypoxic environment since stress can unleash specific responses that could exacerbate cellular deficits. We found that while the rate of proliferation significantly decreased for HC-NPCs under hypoxia, it stayed the same for BD-NPCs, resulting consequently in similar rates of proliferation between HC- and BD-NPCs (Fig. 4). This suggests that either the BD-NPCs were less susceptible to cellular stress, or effect in proliferation cannot be observed after addition of stressors due to the intrinsic stress the BD-NPCs are already under. While the literature has not directly connected resilience to hypoxia with BD, hypoxia is connected to the Wnt/ß-catenin pathway via TLX expression and hypoxia-inducible factor 1 [69, 70]. This pathway activates neurogenesis in NPCs, including proliferation [71]. One possible explanation for this stress resilience is the reduced capacity for STIM2 puncta formation in the BD-NPCs. Loss of STIM2 is known to promote apoptosis resistance by reducing SOCE-dependent Ca^2+^ influx [72]. Additionally, mice with neuron-specific deficiencies in STIM2 are protected from apoptosis under oxidative stress, which is elevated during hypoxia [63]. These data support that a reduction of SOCE from decreased STIM2 expression is neuroprotective under hypoxia. It could be that a reduction in STIM2 puncta formation elicits a similar protection. Importantly, any changes in rate of proliferation could not be due to increased cell death in the cell populations, as a TUNEL assay showed no significant differences in rate of cell death between any of the above conditions (Fig. S6).

Exploring more differentiation-associated phenotypes, neurospheres generated by BD-NPCs were significantly smaller in size with significantly longer neurites, further supporting a trend towards differentiation and away from a symmetric progenitor proliferation ([25]; Fig. 5). The elongated neurites phenotype is consistent with research suggesting that CRMP2, a GSK3β-dependent microtubule-associated protein involved in axonal growth, could be responsible [73]. Transcripts of this protein are not expected to differ as its mechanism of action depends on the ratio of pCRMP2:CRMP2, which is why our RNA-seq did not yield significant differences in this transcript. In addition to decreased size and increased neurite outgrowth, migration was also affected by condition: untreated BD-NPCs migrated farther away from the neurosphere body compared to HC-NPCs and YM58483-treated BD-NPCs. Migration is partly regulated in cells by stromal cell-derived factor 1 (SDF-1, or otherwise known as CXCL12; see [74]). In agreement with our RNA-sequencing data, *CXCL12* was one of the top 3 overexpressed genes in the STAR-DESeq2 analysis (6.7 log_2_-fold change in BD-NPCs, F = 7.7, *p* = 1.86 × 10^−11^), suggesting a role of *CXCL12* for enhanced migration in the BD-NPCs. In addition to speed of migration, another recent study has shown that BD-NPCs have quasi-Brownian migration patterns instead of the typical linear migration patterns in a 2D cell culture model [75]. This further highlight aberrant migration patterns in BD-NPCs.

Some parameters are unable to be probed for in 2D cell culture. Neurogenesis and the development of the human brain is an extremely complex process in which spatio-temporal factors are crucial [12]. To model BD brain growth in a 3D model, cerebral organoids were generated from both HC- and BD-iPSCs and differences in protein expression were analyzed using immunohistochemistry. While testing for the effects that SOCE inhibition has on development, no differences were observed between untreated and treated organoids (Fig. S7). It has been shown, however, that penetrance of certain compounds into organoids is weak [76]. So, the lack of significance may be due to inadequate treatment across the entire organoid. In the future, the generation of sliced neocortical organoids or germline deletion of SOCE effectors STIM1 and STIM2 may uncover a significant role for SOCE in organoid development [46]. The BD organoids, on the other hand, had a reduced subventricular width and area (Fig. 6). This can be the result of an excess of asymmetric divisions which reduce the stem cell pool in the area [12]. Connecting this claim to the reduced capacity of SOCE in our BD-NPCs, Domenichini and colleagues found that pharmacological inhibition of SOCE impaired the self-renewal capabilities of NPCs and shifted cell division to a more asymmetric paradigm, therefore reducing the stem cell population [60]. All of this evidence suggests that BD-NPCs differentiate prematurely.

SOCE was inhibited by blocking ORAI channels with YM throughout all experiments, however this never mimicked the phenotypes observed in the BD-NPCs as we predicted it would. From Fig. 2, it is apparent that reduced capacity for puncta formation is the reason for reduced Ca^2+^ influx, not reduced influx from the ORAI channel. This difference in the method used to block SOCE could explain why we could not replicate the phenotype. Alternatively, SOCE attenuation may be occurring independently of the accelerated differentiation shown here. A closer investigation into the functional impact of attenuated Ca^2+^ influx from SOCE is needed to elucidate this further.

Aside from transcriptional regulation of neurodevelopmental genes, these processes can also be regulated post-transcriptionally by miRNAs. To examine if these types of noncoding RNAs could play a role in the phenotypes shown in our study, we probed for differentially expressed transcripts in an unbiased manner with NanoString technology. From this we found several miRNAs of interest that have differential expressions between HC- and BD-NPCs, namely the *let-7* family of miRNAs. Most interestingly, one of the top predicted targets of *let-7a* according to the online database miRDB (www.mirdb.org; [77]) is *STIMATE*—a mRNA for the STIMATE protein active at the ER and plasma membrane junction where it regulates STIM-ORAI signaling [49, 78]. Although we found no difference in the expression of *STIMATE* mRNA between HC- and BD-NPCs, the possibility exists that translational repression of the protein due to *let-7* miRNAs could help explain the aberrant formation of STIM2 puncta we measure in this study [79]. Beyond this connection, the *let-7* family of miRNAs is also known to regulate neuronal differentiation: increased expression of *let 7a, let-7b*, and *let-7d* promote cell cycle exit by repressing proliferative and cell cycle progression genes such as *TLX and CCND1*, which is consistent with the reduced proliferation in our BD-NPCs and smaller SVZs in the organoids [80–83]. The family is also hypothesized to contribute to an intrinsic clock that regulates the balance between proliferation and differentiation [83]. Overall, we speculate that the overexpression of *let-7* in the BD-NPC lines contributes to the premature differentiation phenotype due to the dysregulation of this intrinsic clock.

Several other miRNAs were upregulated over the late stages of differentiation, one of these being miR-34a. Bavamian and colleagues have similarly found that miR-34a is overexpressed in BD patient postmortem cerebellum tissue and, more interestingly, in BD-NPCs from separate patient lines than ours [26]. Interestingly, miR-34a is known to decrease SOCE activity in human Jurkat T-cells, though in part through STIM1 downregulation [84]. No miRNAs have thus far been identified to target STIM2, though miRNA expression may further contribute to the attenuated Ca^2+^ influx shown here.

In conclusion, we discovered a deficit of SOCE activity in BD neurons and subsequently uncovered several fundamental differences compared to HC-NPCs including a unique transcriptome and miRNA pool, reduced proliferation, enhanced differentiation, and unique 3D development. These data open avenues into exploring BD as a neurodevelopmental disorder and upon further research may elucidate novel mechanism susceptible to environmental factors molecular underpinnings of this complex disease.

### Resource availability

Further information and requests for resources and reagents should be directed to and will be fulfilled by the lead contact Jasmin Lalonde (jlalon07@uoguelph.ca)

### Supplementary information

Supplemental Information

Supplementary Dataset 1

Supplementary Dataset 2.

## Data Availability

Any additional information required to reanalyze the data reported in this paper is available form the lead contact upon request.
