# Supplementary material for: Bipolar disorder-iPSC derived neural progenitor cells exhibit dysregulation of store-operated Ca2+ entry and accelerated differentiation
Source: Mol Psychiatry. 2023 Jul 4;28(12):5237–50. doi: 10.1038/s41380-023-02152-6 (PMC11041787; doi:10.1038/s41380-023-02152-6)
Supplement: Supplementary file 1 — Supplemental Information [file 41380_2023_2152_MOESM1_ESM.pdf]

Supplemental Information for:

Bipolar disorder-iPSC derived neural progenitor cells exhibit dysregulation of store-operated Ca^{2+} entry and accelerated differentiation

Tristen Hewitt[1,5,6], Begüm Alural[1,6], Manali Tilak[1], Jennifer Wang[2], Natalina Becke[1], Ellis Chartley[1], Melissa Perreault[3], Stephen J Haggarty[2], Steven D Sheridan[2], Roy H Perlis[2], Nina Jones[1], Nikolaos Mellios[4], Jasmin Lalonde[1*]

[1] Department of Molecular and Cellular Biology, University of Guelph, Guelph, ON, Canada

[2] Center for Quantitative Health, Center for Genomic Medicine and Department of Psychiatry, Massachusetts General Hospital, Boston, MA, 02114, USA

[3] Department of Biomedical Sciences, Ontario Veterinary College, University of Guelph, Guelph, ON, Canada

[4] Department of Neurosciences, University of New Mexico School of Medicine, Albuquerque, NM, USA

[5] Present address: Neuroscience Center, HiLIFE, University of Helsinki, Helsinki, Finland

[6] These authors contributed equally to this work.

* To whom correspondence should be addressed: jlalon07@uoguelph.ca

SUPPLEMENTARY MATERIAL AND METHODS

Cell culture

6-well culture plates were coated with Geltrex (Thermo Fisher Scientific, Waltham, MA) and stored at 4°C for up to 2 weeks. Plates were warmed at RT for 1 hour prior to seeding. NPCs were lifted using Accutase (Millipore Sigma, St. Louis, MO) and were seeded at 5.0 x 10^5 NPCs per well unless otherwise stated. NPCs were cultured in Neural Expansion Medium containing 49% Neurobasal, 49% Advanced DMEM/F12 and 2% Neural Induction Supplement (NEM; Gibco) at 37°C and 5% CO_2.

Fluo-4 Ca^{2+} imaging

HC and BD iPSC/NPCs were seeded (5.0 x 10^5 cells) onto Geltrex-coated 35 mm glass-bottom dishes (MatTek, Ashland, MA) 24 h prior to imaging, or differentiated for 2 and 8 weeks prior to imaging. Cells were loaded with 2 µM of the Ca^{2+} sensitive acetoxymethyl ester (Fluo-4, AM; Thermo Fisher Scientific) diluted in an imaging solution containing 10 mM HEPES pH 7.3, 140 mM NaCl, 5 mM KCl, 2 mM $CaCl_2$, 1 mM $MgCl_2$, and 10 mM glucose for 45 minutes. The cells were then washed and rested in the Ca^{2+}-containing HEPES imaging solution for 20 minutes. They were then washed in a Ca^{2+}-free HEPES imaging solution containing 10 mM HEPES pH 7.3, 140 mM NaCl, 5 mM KCl, 3 mM $MgCl_2$, and 10 mM glucose and incubated in 2 µM Thapsigargin (Tg; MilliporeSigma, Burlington, MA) diluted in the Ca^{2+}-free HEPES solution for 10 minutes. For neurons differentiated for 2 or 8 weeks, a cocktail of inhibitors for L-type Ca^{2+} channels (10 µM nimodipine), AMPA/kainite receptors (10 µM CNQX) and NMDARs (50 µM APV) were added in addition to the Tg. Imaging was conducted using a Nikon Eclipse Ti2 (Nikon, Minato City, TYO) and a 40× objective. After 3 minutes of baseline fluorescence measurements at 1 frame

every 5 seconds, the Ca^{2+}-free HEPES solution was replaced with the Ca^{2+}-containing HEPES imaging solution with 2 μM Tg. For TRPC4/5 and NMDA inhibition, the imaging solutions with 10 μM ML 204 (Tocris), 10 μM AC 1903 (Tocris), and 50 μM APV (Tocris), sequentially. 5 minutes of imaging was conducted after each treatment. All experiments were performed at room temperature. Image processing and analysis was done using NIS Elements (Nikon, Minato City, TYO) and Microsoft Excel.

Fura-2 Ca^{2+} imaging

For ratiometric Ca^{2+} imaging, iPSC/NPCs were incubated in imaging solution with 2 μM of the Fura-2 AM dye (Thermo Fisher Scientific) similar to the Fluo-4 protocol but without addition of Tg after the last wash. Intracellular Ca^{2+} concentration ($[Ca^{2+}]_i$) was estimated according to the following standard equation (1):

$$[Ca^{2+}]_i = QK_d\,(R - R_{min}) \;/\; (R_{max} - R)$$

In this equation, R is the F_{340}/F_{380} fluorescence ratio while an in situ Fura-2 calibration session was done to estimate the values of R_{min} and R_{max}. Specifically, R_{min} and R_{max} were calculated from the fluorescence ratios with imaging solutions containing 1 mM EGTA + 2 μM ionomycin and 20 mM Ca^{2+} + 2 μM ionomycin, respectively. For its part, Q was determined from the F_{min}/F_{max} ratio at 380 nm and K_d represents the apparent dissociation constant of Fura-2 binding to Ca^{2+} (135 nM). In our hands, the values of these parameters were: $R_{min} = 0.225 \pm 0.001$, $R_{max} = 1.275 \pm 0.009$, and $Q = 2.08$.

Puncta formation assay

iPSC/NPCs (5.0 x 10^5 cells) were seeded onto Geltrex-coated 35 mm glass-bottom dishes (MatTek) 1 day prior to transfection with Lipofectamine 2000 (Thermo Fisher Scientific) as per the manufacturer's protocol. 48 hours after transfection the cells were incubated with 2 µM Tg for 10 minutes prior to imaging using a Nikon Eclipse Ti2 microscope. Image analysis, including total puncta count and cell area measurements, were conducted in ImageJ (NIH, Rockville, MD).

Seahorse mitochondrial stress test

To evaluate mitochondrial function in HC-iPSC/NPCs compared to BD-iPSC/NPCs, an XF Mito Stress Test was performed using the Agilent Seahorse XF Analyzer (Santa Clara, CA) as per the manufacturer's protocol. Media was made fresh using the Seahorse XF Base DMEM with added 2 mM L-glutamine, 10 mM glucose and 1 mM sodium pyruvate (bicarbonate-free). 1 day before 1.5 x 10^5 cells/well for each NPC line were seeded onto 20 wells of a Geltrex-coated 24-well XF cell culture microplate (Seahorse Bioscience). The calibration plate was hydrated in a non-CO_2 37ºC incubator overnight. Basal oxygen consumption rate (OCR) and extracellular acidification rate (ECAR) were recorded 4 times in 20 mins, after which 3 different compounds were added sequentially (2 µM oligomycin, 2 µM FCCP and 0.5 µM antimycin A/rotenone) to measure the 4 fundamental parameters of mitochondrial function and their derivatives: basal respiration, maximal respiration, ATP production, spare respiratory capacity and coupling efficiency. At the end of the experiment run, cell densities from each well were quantified by using crystal violet staining and used for normalization of the results.

EdU assay

The Click-iT EdU Cell Proliferation Kit for Imaging (Invitrogen) was used as per the manufacturer's protocol. In brief, NPCs were plated onto Geltrex-coated 24 well plates with coverslips and incubated for the prescribed amount of time (6 and 12 hours) under normaxia (humidified chamber; ambient O_2, 5% CO_2, and 37ºC) and hypoxia (HypOxystation H35 [HypOxygen, Frederick, MD]; 1% O_2, 5% CO_2, and 37ºC) in 10 µM 5-ethynyl-2'-deoxyuridine (EdU). Importantly, EdU was added using a half media change (20 µM EdU in NEM) to avoid disturbing cell cycle kinetics. After incubation cells were fixed with 4% PFA for 15 minutes. Cells were then washed with PBS + 3% bovine serum albumin (BSA) and permeabilized with 0.5% Triton X-100 in PBS for 20 minutes. After another PBS + 3% BSA wash cells were incubated in the Click-iT reaction cocktail containing 86% Click-iT reaction buffer, 4% $CuSO_4$, 0.24% Alexa Fluor-594 azide, and 10% reaction buffer additive for 30 minutes protected from light. The cells were washed with PBS + 3% BSA and stained with 4',6-diamidino-2-phenylindole (DAPI) for 15 minutes before mounting the coverslips on glass slides with ProLong Diamond Antifade.

Images were captured on a Nikon Eclipse Ti2 microscope using the NIS Elements software. Analysis was conducted using ImageJ (NIH) as follows. After a threshold fluorescence was set the image was made binary and watershed was used to separate clumped cells. Particles between 150 and 5,500 pixels were analyzed for a final count of total and EdU positive cells. Rate of proliferation was measured using EdU positive cells over total cell count.

TUNEL assay

The Roche *In Situ* Cell Death Detection Kit (Millipore Sigma) was used as per the manufacturer's protocol to assay cell viability between HC- and BD-NPCs under normoxia (ambient O_2, 5% CO_2,

and 37ºC) and hypoxia (1% O_2, 5% CO_2, and 37ºC). Briefly, after treatment, cells were fixed using 4% PFA for 20 minutes. After permeabilization with 0.1% Triton-X for 2 mins, the cells were incubated in TUNEL reaction solution for 30 mins in the dark at 37ºC with humidity. After a PBS rinse, cells were incubated in DAPI staining solution before being mounted onto slides for visualization as above.

Neurosphere assay

iPSC/NPCs were seeded at 1.0 x 10^4 in ultra-low adhesion U-bottom 96 well plates (Corning) and were agitated at 75 rpm in NEM for 72 hours. Neurospheres were then transferred to Geltrex-coated 24-well plates and incubated in differentiation media for 72 hours. Phase contrast images were collected on a Nikon Eclipse Ti2 microscope before fixing the neurospheres in 4% paraformaldehyde (PFA) for 20 minutes.

Immunocytochemistry

Immunocytochemistry was carried out for neurospheres and 2D NPC cultures grown on Geltrex-coated coverslips in 24-well plates as previously described (2, 3). Imaging was done with a Nikon Eclipse Ti2 microscope and processing was conducted using ImageJ (NIH).

Immunoblotting

Cell protein lysates and immunoblotting were performed as previously described (3). For quantification of immunoblot data, the intensity of each band of interest was divided by the intensity of its respective loading control (GAPDH) to provide the normalized expression value used for statistical analysis.

RNA-sequencing

RNA was collected from each of the 6 cell lines at the NPC stage as follows. Approximately 1.0-1.5 x 10^6 NPCs were harvested by manual scraping and pellets collected by centrifugation. Total RNA was extracted from cell pellets using the Qiagen miRNeasy mini kit with a 30 µL RNase free water elution. Agilent Bioanalyzer QC was performed on a 1:100 dilution of the stock RNA using the Pico RNA chip. RNA samples with RIN scores greater than 7 were diluted to 40 ng/µL using concentrations measured with the Qubit HS RNA assay (Invitrogen). Sequencing libraries were prepared with the KAPA Stranded RNA-Seq Kit with RiboErase and sequenced on Illumina Hiseq4000 to achieve ~80M paired end, 100 base pair reads for each sample.

Quality control of the paired-end reads was done with FastQC (v0.11.9) and adaptor trimming using BBduk (v39.00). To account for bias in using a single publicly available RNA-seq data analysis package, we analyzed our results sequentially using multiple packages. First, reads were mapped onto the human genome (GRCh38) using Hisat2 v2.0.5 and counted using FeatureCounts v1.6.2 default parameters. Differential expression analysis was performed using the DESeq2 v1.6.3 package. Reads were then mapped onto the hg38.p13 human genome (GENCODE release 41) using STAR aligner (v2.7.10a with the 'alpha_220207' patch). Here, differential expression analysis was performed using the DESeq2 (v1.34.0) and edgeR (v3.36.0) packages. Genes were labelled as significantly altered if adjusted p-value ≤ 0.05 and $\log_2$ fold change ≥ 2 or ≤ -2. The differentially expressed transcripts were then grouped by biological process using the GeneOntology online resource. Specific statistics can be found in the figure caption and in Supplementary File 1.

Cerebral organoid generation and staining

For preparation of cerebral organoids, iPSCs of all 6 cell lines were maintained in culture on rLaminin-521 (Corning, 354222) with Nutristem XF/FF Medium (Biological Industries, 05-100-1A). Cerebral organoids were generated from the iPSCs using the STEMdiff Cerebral Organoid Kit), according to the manufacturer's protocol (StemCell Technologies, Vancouver, BC). Briefly, embryoid bodies (EBs) were formed from 9 x 10^3 iPSCs per/well by incubation with EB Seeding Medium in 96-well round-bottom ultra-low attachment plate (Corning, 7007) at 37ºC for 5 days. On the day 5, Induction Medium was added and EBs were transferred to a 24-well plate and incubated for 2 days at 37ºC. On the day 7, EBs were transferred into Matrigel droplets and incubated in Expansion Medium at 37ºC for 3 days. On the 10th day, media was changed to Maturation Medium and the organoids were allowed to mature for 40-50 days at 37ºC on an orbital shaker at 65 rpm. On day 20, 3 µM YM58483 was added to select organoids for a 20 days chronic treatment. Once the organoids had reached maturity (day 40/50), they were frozen in a gelatin/sucrose solution for slicing. Organoids were sliced using a Leica CM1860 cryostat at a thickness of 20 µm. Sections were then stained according to the immunocytochemistry procedure described above and imaged using a Nikon Eclipse Ti2 microscope.

NanoString miRNA profiling

The nCounter Human miRNA Expression Assay Kit v3 (Nanostring Technologies, Seattle, WA, USA) was used for miRNA profiling. This expression panel contains 799 probes that represent >95% of all human miRBase reads. RNA was collected as above from the six cell lines in addition to two others, GM08330 and GM05440, described previously (4). Briefly, 100 ng of total RNA was annealed to the nCounter miRNA Tag reagent, hybridized to the Reporter CodeSet, and run

on an nCounter Prep Station. The nSolver analysis software (NanoString Technologies) was used for normalization and analysis. Normalization was done by using the geometric mean of all miRNAs and controlling for negative control signals.

Real-time quantitative PCR for miR-34a

Expression levels of miR-34a and housekeeping miRNAs (RNU6B and SNORD_95) were determined with predesigned TaqMan assays (Life Technologies, Foster City, CA). High-Capacity cDNA synthesis Kit with RNase inhibitor (Thermo Fisher Scientific) was used to synthesize cDNA from 500 ng of total RNA. RT-qPCR experiments were performed on a LightCycler®480 Instrument (Roche, Germany) according to the manufacturer's instructions. miR-34a expression level in each sample was normalized to the geometric mean of RNU6B and SNORD_95 expressions. The $2^{-\Delta\Delta}C_T$ method (5) was used to determine relative miRNA expression levels.

Statistics

All statistical figures are presented as mean ± standard error of mean (SEM) unless otherwise stated. After testing for normality with Levene's test and equal variance with a Shapiro-Wilk test, the appropriate analysis was carried out. Comparison between 2 groups used two-tailed Student's *t*-test with Welch's correction when variance was unequal. Multiple comparisons were analyzed using an ANOVA when assumptions of normality and equal variance were met. The Mann-Whitney U test or Kruskal-Wallis test was used when these assumptions were violated. Any p-value of less than 0.05 was considered to be significant. All analysis was conducted using R. Specific statistical methods are described in the figure legends.

SUPPLEMENTARY METHODS REFERENCES

1. Grynkiewicz G, Poenie M, Tsien RY. A new generation of Ca^{2+} indicators with greatly improved fluorescence properties. J Biol Chem. 1985;260:3440–3450.

2. Lalonde J, Saia G, Gill G. Store-operated calcium entry promotes the degradation of the transcription factor Sp4 in resting neurons. Sci Signal. 2014;7(328):ra51.

3. Lalonde J, Reis SA, Sivakumaran S, Holland CS, Wesseling H, Sauld JF, et al. Chemogenomic analysis reveals key role for lysine acetylation in regulating Arc stability. Nat Commun. 2017;8(1):1659.

4. Bavamian S, Mellios N, Lalonde J, Fass DM, Wang J, Sheridan SD, et al. Dysregulation of miR-34a links neuronal development to genetic risk factors for bipolar disorder. Mol Psychiatr. 2015;20(5):573–84.

5. Livak KJ, Schmittgen TD. Analysis of relative gene expression data using real-time quantitative PCR and the 2(–Delta Delta CC(T)) method. Methods. 2001;25:402–408.

SUPPLEMENTARY FIGURES AND DATASET

Figure S1. HC-NPC and neuronal validation after iPSC induction and differentiation.

Figure S2. Patient- and cell-specific Ca^{2+} influx measures.

Figure S3. Disparity in SOCE persists through blocking TRPC4/5 and NMDARs.

Figure S4. Example image showing distribution of STIM1 puncta before and after Tg treatment.

Figure S5. Mitochondria function normally in BD-NPCs.

Figure S6. TUNEL assay demonstrates no difference in rates of cell death between HC- and BD-NPCs under normoxic and hypoxic conditions.

Figure S7. HC organoids grown under SOCE inhibition do not have developmental abnormalities.

Figure S8. qPCR quantifying miR-34a levels in HC- and BD-NPCs and neurons over differentiation.

Supplementary Dataset 1. RNA-sequencing comparing the transcriptome of HC- and BD-NPCs.

Supplementary Dataset 2. Nanostring miRNA analysis in BD-NPCs and neurons compared to HC-NPCs and neurons differentiation for 2, 4, and 6 weeks.

A

Cell line identifier	Experimental condition / Lithium responsiveness	Sex	Age (years)
PSC-01-024	Healthy control	Female	36
PSC-01-185	Healthy control	Female	37
PSC-01-223	Healthy control	Male	45
PSC-01-003	BD - lithium responder	Male	51
PSC-01-009	BD - lithium non-responder	Male	52
PSC-01-122	BD - lithium non-responder	Female	49

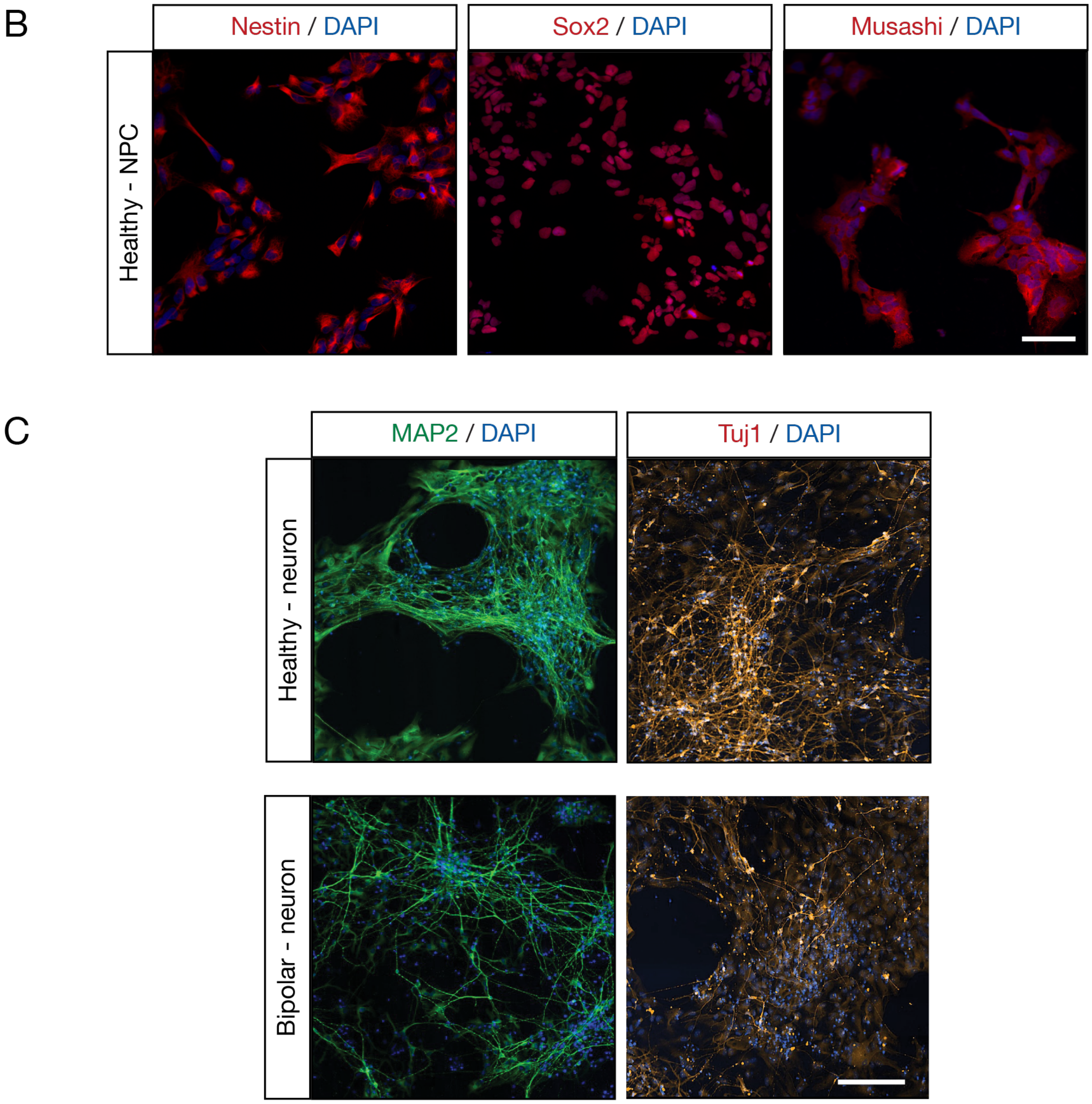

Figure S1. HC-NPC and neuronal validation after iPSC induction and differentiation. (A) Fibroblasts were collected through skin punch from 6 individuals, 3 of which were healthy controls (Cell line identifiers PSC-01-024/185/223) and 3 of which who were diagnosed with type I BD (Cell line identifiers PSC-01-003/009/122). These fibroblasts were reprogrammed with mRNA to iPSCs and then subsequently re-differentiated into NPCs. Of note, 1 of the BD patients was responsive to the mood stabilizer lithium while the other 2 were unresponsive. Due to this, the effects of lithium cannot be addressed in this research. (B) Representative fluorescent images from one HC-NPC line (PSC-01-024) outlining the expression of three markers of NPCs Nestin (left), Sox2 (middle) and Musashi (right). Nucleus is visualized with DAPI in all three micrographs. NPC status was confirmed for all other lines using the same staining. Scale bar represents 50 µm. (C) The differentiation capabilities of the NPCs to differentiate into mature neurons was validated with staining of MAP2 and Tuj1 in healthy (representative image PSC-01-223) and BD (representative image PSC-01-003) lines up to and including 6 weeks of maturation. Scale bar represents 30 µm.

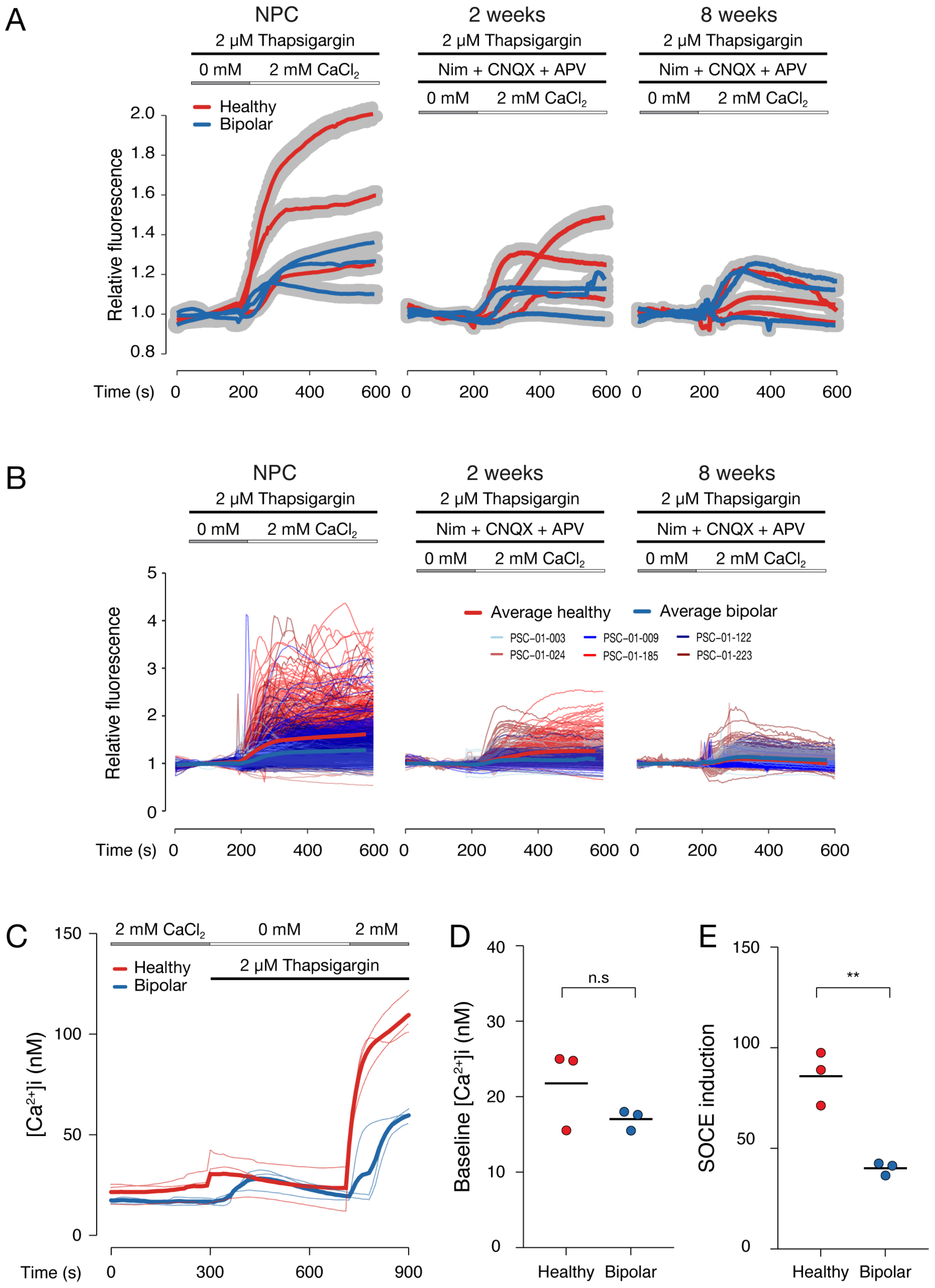
A
NPC
2 µM Thapsigargin
0 mM
2 mM $CaCl_2$
2 weeks
2 µM Thapsigargin
Nim + CNQX + APV
0 mM
2 mM $CaCl_2$
8 weeks
2 µM Thapsigargin
Nim + CNQX + APV
0 mM
2 mM $CaCl_2$
Healthy
Bipolar
Relative fluorescence
2.0
1.8
1.6
1.4
1.2
1.0
0.8
Time (s)
0
200
400
600
B
NPC
2 weeks
8 weeks
Average healthy
Average bipolar
PSC-01-003
PSC-01-009
PSC-01-122
PSC-01-024
PSC-01-185
PSC-01-223
Relative fluorescence
5
4
3
2
1
0
Time (s)
C
2 mM $CaCl_2$
0 mM
2 mM
2 µM Thapsigargin
Healthy
Bipolar
$[Ca^{2+}]i$ (nM)
150
100
50
0
Time (s)
0
300
600
900
D
Baseline $[Ca^{2+}]i$ (nM)
40
30
20
10
0
n.s
Healthy
Bipolar
E
SOCE induction
150
100
50
0
**
Healthy
Bipolar

Figure S2. Patient- and cell-specific Ca^{2+} influx measures. (A) Relative (Fluo-4) Ca^{2+} imaging from Figure 1 was separated by individual cell line, showing average increase in fluorescence for each of the 3 HC and BD-NPCs and neurons after 2 and 8 weeks of differentiation. Data is presented as mean (red and blue curve) ± s.e.m (grey ribbon). (B) Fluo-4 Ca^{2+} imaging from Figure 1 showing curves from each cell in all 6 cell lines, including PSC-01-003 (light blue), PSC-01-009 (blue), PSC-01-122 (dark blue), PSC-01-024 (red), PSC-01-185 (light red), and PSC-01-223 (dark red). The same average shown in Figure 1 is given on top of these curves for HC- (red) and BD- (blue) NPCs and neurons differentiated for 2 and 8 weeks. (C-E) The experimental results at the NPC stage were confirmed with ratiometric Fura-2 imaging using all 6 cell lines (PSC-01-024: n = 76; PSC-01-185: n = 80; PSC-01-223: n = 97; PSC-01-003: n = 51; PSC-01-009: n = 110; PSC-01-122: n = 60). (D) Baseline concentrations (0-180s) show no differences between HC- and BD-NPCs (t_4 = 1.475, p = 0.21). (E) After acquiring baseline values, Tg was added and Ca^{2+} was removed from the media, followed by the re-addition of Ca^{2+} at 720s. Ca^{2+} influx was measured as the difference between fluorescence at 900s and 720s. HC-NPCs had significantly higher Ca^{2+} influx than BD-NPCs (t_4 = 5.750, p = 0.0045).

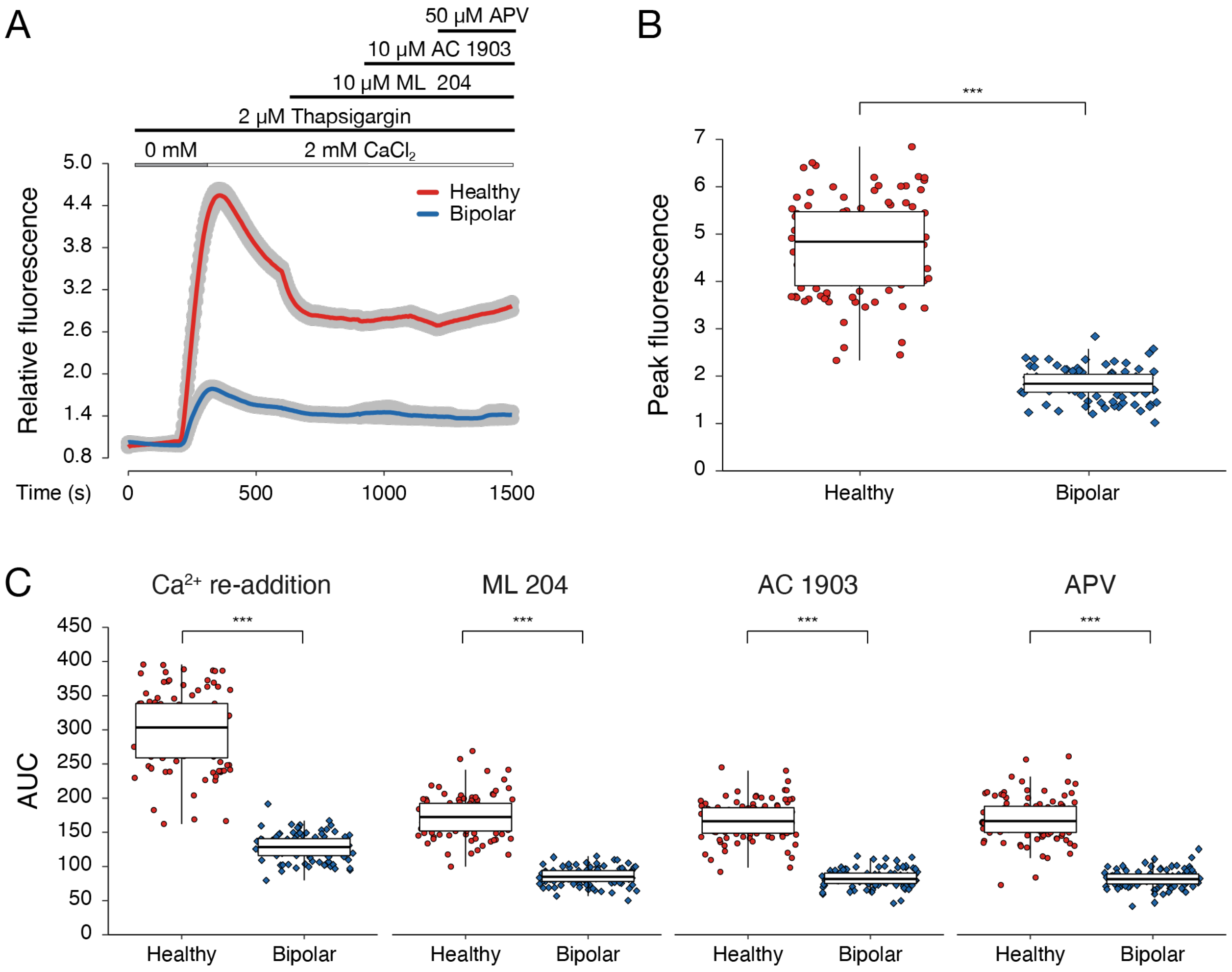

Figure S3. Disparity in SOCE persists through blocking TRPC4/5 and NMDARs. (A) Ca^{2+} imaging was performed as above for 10 minutes, followed by exchange with media containing 10 μM ML 204, 10 μM AC 1903, or 50 μM APV for 5 minutes each. Data is presented as mean (red and blue curve) ± s.e.m (grey ribbon). Measurements were conducted on 100 cells from 1 HC line (PSC-01-223) and 99 cells from 1 BD (PSC-01-003). (B) Peak overall fluorescence during imaging. HC-NPCs had significantly higher peak fluorescence ($W = 12$, $p < 0.001$) than BD-NPCs. (B) AUC analysis of SOCE specific Ca^{2+} influx, and decreases after inhibition with the Ca^{2+} channel blockers. HC-NPCs had significantly greater AUCs than BD-NPCs after Ca^{2+} re-addition ($W = 0.88$, $p < 0.001$), after blocking TRPC4 with ML 204 ($W = 0.92$, $p < 0.001$), after

blocking TRPC5 with AC 1903 (W = 0.92, $p < 0.001$), and after blocking NMDA with APV (W = 0.92, $p < 0.001$). (C) Peak overall fluorescence during imaging. HC-NPCs had significantly higher peak fluorescence (W = 12, $p < 0.001$) than BD-NPCs.

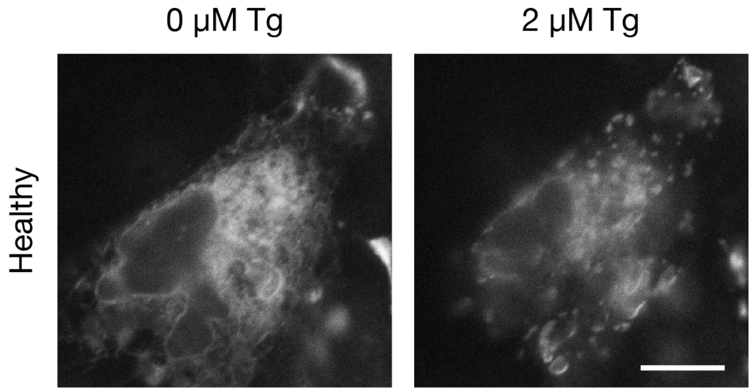

Figure S4. Example image showing distribution of STIM1 puncta before and after Tg treatment. Before depleting ER stores with Tg, STIM1 has a qualititaive dispersed distribution in the cell. After 10 minutes of 2 µM Tg treatment, this distribution shifts to punctate. Scale bar represents 10 µm.

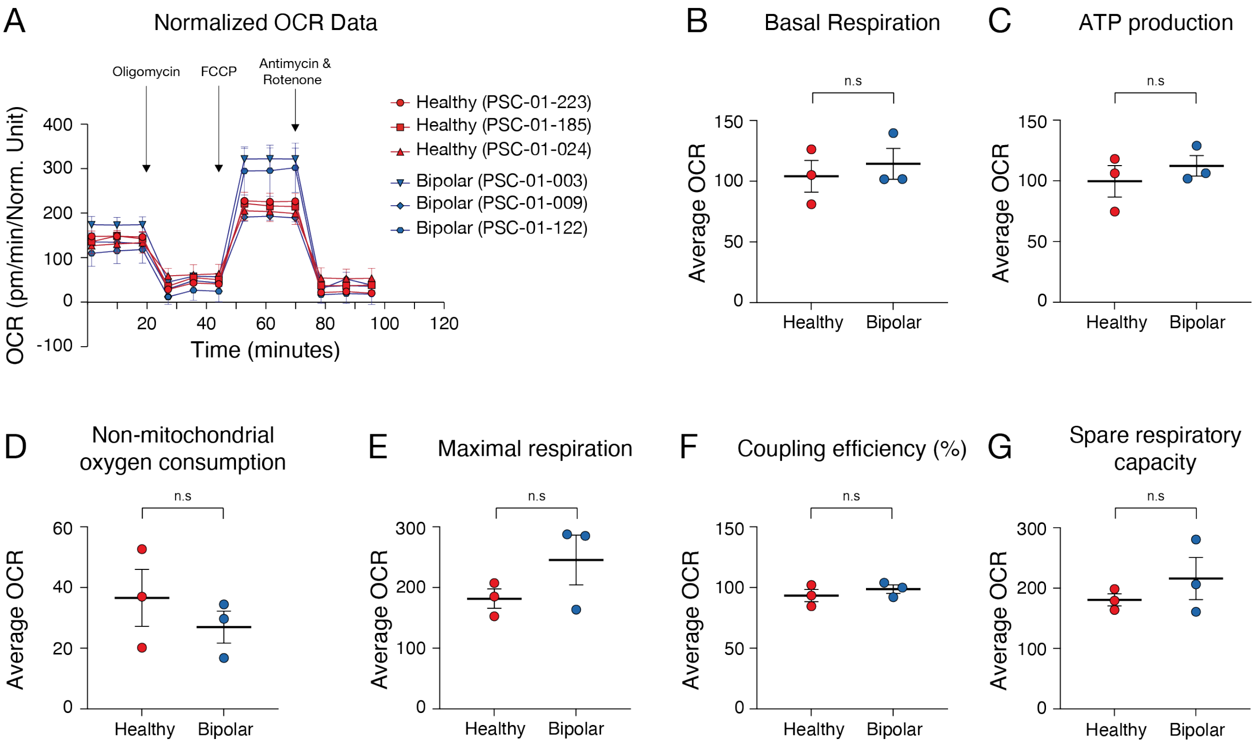

Figure S5. Mitochondria function normally in BD-NPCs. (A) A Seahorse mitochondrial stress test was performed to measure the following parameters between HC-NPCs and BD-NPCs (*n* =3): (A) the real-time kinetic rate of oxygen consumption (OCR) and indication of the injection time of the compounds, (B) basal respiration (t_4 = 0.5620, *p* =0.6041), (C) ATP production (t_4 = 0.8225, *p* = 0.4570), (D) non-mitochondrial respiration (t_4 = 0.8966, *p* = 0.4206), (E) maximum respiration capacity (t_4 = 1.452, *p* = 0.2201), (F) coupling efficiency (t_4 = 0.8670, *p* = 0.4348), and (G) spare respiratory capacity (t_4 = 0.9724, *p* = 0.3859). As no significance was found between HC-NPCs and BD-NPCs for any of these parameters, it can be concluded that mitochondria function is consistent between these two groups. Data is presented as mean ± s.e.m. n.s signifies $p > 0.05$; Student's *t*-test.

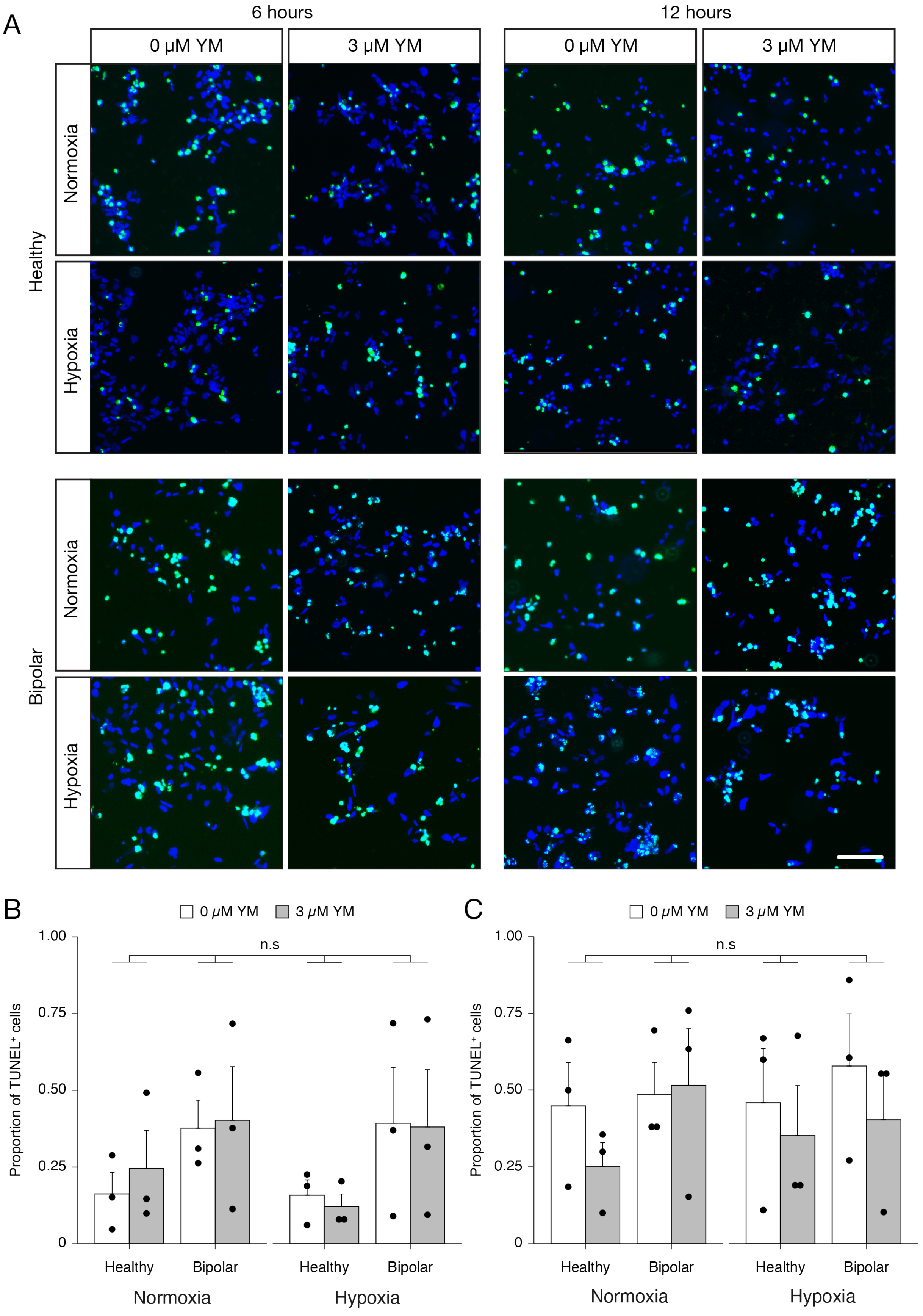
A
6 hours
12 hours
0 μM YM
3 μM YM
0 μM YM
3 μM YM
Healthy
Normoxia
Hypoxia
Bipolar
Normoxia
Hypoxia
B
0 μM YM
3 μM YM
n.s
Proportion of TUNEL+ cells
1.00
0.75
0.50
0.25
0
Healthy
Bipolar
Healthy
Bipolar
Normoxia
Hypoxia
C
0 μM YM
3 μM YM
n.s
Proportion of TUNEL+ cells
1.00
0.75
0.50
0.25
0
Healthy
Bipolar
Healthy
Bipolar
Normoxia
Hypoxia

Figure S6. TUNEL assay demonstrates no difference in rates of cell death between HC- and BD-NPCs under normoxic and hypoxic conditions. (A) Representative immunofluorescent images of HC- and BD-NPCs without and with SOCE inhibition via 3 µM YM58483 after 6 and 12 hours of incubation under normoxic and hypoxic conditions with double-stranded DNA breaks visualized using TUNEL (green). DAPI is shown in blue. Scale bar represents 100 µm. (B, C) Quantification of $TUNEL^{+}$ cells / DAPI after 6 and 12 hours of incubation under normoxia and hypoxia to measure rate of cell death. No significant interaction effect was present for either 6 hours ($F_{3, 20} = 2.4$, $p = 0.094$, $n = 6$) or 12 hours ($F_{3,20} = 0.51$, $p = 0.68$, $n = 6$). When testing main effects, BD-NPCs had significantly higher rates of cell death at 6 hours ($F_{1,21} = 7.3$, $p = 0.013$, $n = 12$), but this did not persist after 12 hours ($F_{1,21} = 1.5$, $p = 0.23$, $n = 12$). Model testing was used to determine a lack of evidence to include YM58483 treatment to the model, and so results at 0 µM and 3 µM YM58483 treatment were pooled for both the 6- and 12-hour analyses. Data is presented as mean ± s.e.m. n.s signifies $p > 0.05$; ANOVA with Tukey's post hoc test when appropriate.

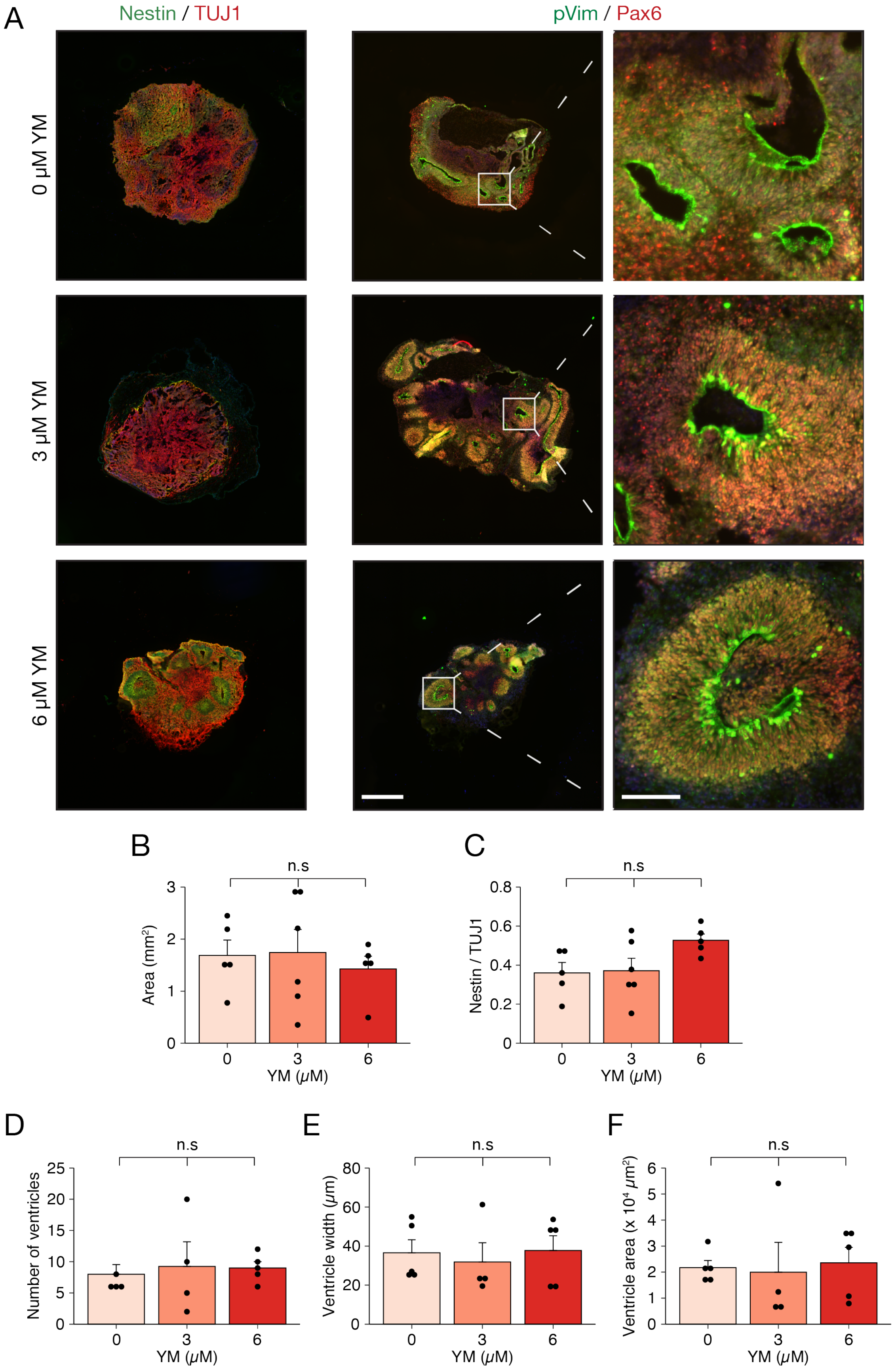
A
Nestin / TUJ1
pVim / Pax6
0 μM YM
3 μM YM
6 μM YM
B
n.s
Area (mm²)
YM (μM)
C
n.s
Nestin / TUJ1
YM (μM)
D
n.s
Number of ventricles
YM (μM)
E
n.s
Ventricle width (μm)
YM (μM)
F
n.s
Ventricle area (x 10⁴ μm²)
YM (μM)

Figure S7. HC organoids grown under SOCE inhibition do not have developmental abnormalities. (A) Micrographs of a representative HC cell line matured to 40 days without or with 20 days exposure to YM58483. Organoids were co-stained for radial glia marker Nestin and immature neuron marker TUJ1 or for migration marker pVim and proliferation marker PAX6. Inlet shows an example SVZ. Scale bars represent 500 µm and 100 µm. (B) Average area of organoids. Significance was not reached for untreated (n = 5) and treated (3 µM YM, n = 6; 6 µM YM, n = 5) organoids ($F_{2,13} = 0.22$, $p = 0.81$). (C) Ratio of Nestin/TUJ1 used to measure organoid maturity. No significant differences were observed ($F_{2,13} = 2.89$, $p = 0.091$). (D) Number of ventricles quantified. Significance was not reached ($F_{2,13} = 0.40$, $p = 0.68$). (E) Area of SVZ quantified. Significance was not reached ($F_{2,11} = 0.068$, $p = 0.94$). (F) Width of ventricle. Significance was not reached ($\chi^2 = 0.78$, df = 2, $p = 0.68$). Data is presented as mean ± s.e.m. n.s signifies $p > 0.05$; ANOVA with Tukey's post hoc test was used for parametric data. Kruskal-Wallis test was used for non-parametric data.

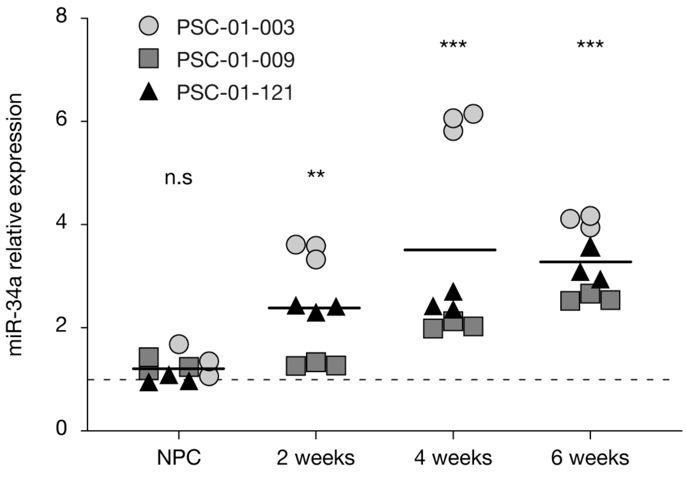

Figure S8. qPCR quantifying miR-34a levels in HC- and BD-NPCs and neurons over differentiation. Cells were differentiated for 2, 4, and 6 weeks before RNA collection. For each line, relative miR-34a expression was calculated by normalizing expression levels to HC-NPCs. Shapes distinguish between cell lines: light grey represent PSC-01-003, dark grey represent PSC-01-009, and black triangles represent PSC-01-121. Where miR-34a expression did not differ between HC- and BD-NPCs (W = 32, $p = 0.49$, $n = 9$), it did after 2 weeks (W = 6, $p = 0.0012$, $n = 9$), 4 weeks (W = 0, $p < 0.001$, $n = 9$), and 6 weeks (W = 0, $p < 0.001$, $n = 9$) weeks of differentiation. Mean of all 9 replicates per differentiation time point is presented as a black bar. n.s signifies $p > 0.05$, ** $p < 0.01$, *** $p < 0.001$; Mann-Whitney U test.

Supplementary Material and Methods

**REAGENT or RESOURCE**	**SOURCE**	**IDENTIFIER**
Antibodies		
Rabbit anti-Stim1 at 1:1000	Alomone	Cat. # ACC-063; RRID:AB_2039893
Rabbit anti-STIM2 at 1:300	Alomone	Cat. # ACC-064; RRID:AB_2040218
Rabbit anti-ORAI1 at 1:300	Alomone	Cat. # ACC-060; RRID:AB_2039844
Rabbit anti-ORAI2 at 1:300	Alomone	Cat. # ACC-061; RRID:AB_2040046
Rabbit anti-ORAI3 at 1:800	Alomone	Cat. # ACC-065; RRID:AB_10915896
Rabbit anti-Nestin at 1:2000	Millipore Sigma	Cat. # ABD69; RRID:AB_2744681
Mouse anti-Tubulin β 3 (TUBB3) at 1:1000	BioLegend	Cat. # 801201; RRID:AB_2313773
Rabbit anti-PAX6 at 1:400	BioLegend	Cat. # 901301; RRID:AB_2565003
Mouse anti-phosphorylated vimentin (Ser55) at 1:1000	MBL Life Sciences	Cat. # D076-3; RRID:AB_592963
Chicken anti-GAPDH at 1:100 000	Millipore Sigma	Cat. # AB2302; RRID:AB_10615768
Chemicals, Peptides, and Recombinant Proteins		
Fluo-4, AM, cell permeant	Invitrogen	Cat. # F14201
Geltrex™ LDEV-Free, hESC-Qualified, Reduced Growth Factor Basement Membrane Matrix	Gibco	Cat. # A14133-02
StemPro™ Accutase™ Cell Dissociation Reagent	Gibco	Cat. # A6964
PSC Neural Induction Medium	Gibco	Cat. # A1647801
Lipofectamine™ 2000 Transfection Reagent	Invitrogen	Cat. # 11668019
Protein Assay Dye Reagent Concentrate	Bio-Rad	Cat. # 5000006
Opti-MEM™ I Reduced Serum Medium	Gibco	Cat. # 31985-062
Critical Commercial Assays		
Click-iT™ EdU Cell Proliferation Kit for Imaging, Alexa Fluor™ 488 dye	Invitrogen	Cat. # C10339
High-Capacity cDNA Synthesis Kit with RNase inhibitor	ThermoFisher	Cat. # 4374966
miRNeasy mini kit	QIAGEN	Cat. # 217004

nCounter Human miRNA Expression Assay Kit v3	Nanostring	Cat. # CSO-MIR3-12
Roche *In Situ* Cell Death Detection Kit	Millipore Sigma	Cat. # 11684795910
Roche Stranded RNA-seq Kit with RiboErase	KAPA	Cat. # 07962142001
STEMdiff™ Cerebral Organoid Kit	StemCell Technologies	Cat. # 08570
STEMdiff™ Cerebral Organoid Maturation Kit	StemCell Technologies	Cat. # 08571
Deposited Data		
RNA-sequencing	This paper	UniProt
Experimental Models: Cell Lines		
PSC-01-024	This paper	N/A
PSC-01-185	This paper	N/A
PSC-01-223	This paper	N/A
PSC-01-003	This paper	N/A
PSC-01-009	This paper	N/A
PSC-01-122	This paper	N/A
GM08330	Bavamian et al., 2015	
GM05440	Bavamian et al., 2015	
